# Heavy-Metal Rankings Across Sediment Risk, Seafood Bioaccumulation and Dietary Hazard in the Maowei Sea, China

**DOI:** 10.3390/toxics14100875

**Published:** 2026-09-30

**Authors:** Enze Zhang, Lijiao Deng, Jun Zeng, Junxiang Lai, Hao Li

**Affiliations:** 1 College of Architecture and Civil Engineering, Beijing University of Technology, Beijing 100124, China; 2 Beijing Zhongdihongke Environmental Technology Co., Ltd., Beijing 100085, China; 3 Guangxi Key Laboratory of Marine Environmental Science, Guangxi Academy of Marine Sciences, Guangxi Academy of Sciences, Nanning 530007, China

**Keywords:** biota-sediment accumulation factor, inorganic arsenic, target hazard quotient, Monte Carlo simulation, mariculture, Beibu Gulf

## Abstract

Sediment indices usually set heavy-metal priorities in mariculture bays, but uptake and regulation vary among taxa, so seafood priorities may differ. In a single-season survey of the Maowei Sea (Beibu Gulf) in April 2022, seven metals were measured in water (27 stations), sediment (15 stations) and seven commercial taxa, each represented by the mean of 12 separately analysed station composites. We compared their rankings across sediment risk, bioaccumulation and dietary hazard. Cd and Hg contributed 85% of the sediment ecological risk index. Biota-sediment accumulation factors were highest for Cd in clams, Zn in oyster, Cu in crustaceans and Hg in fish. At an assumed 10% inorganic-As fraction, dietary hazard indices exceeded 1 for hard clam (1.44) and mitten crab (1.04). At 5%, no taxon exceeded 1. Under both fractions, the leading dietary metal in most taxa was As or Cu, not Cd or Hg. Cd or Hg took over in clams and fish at lower fractions, but not in crustaceans or oyster. In this survey, sediment rankings were a weak guide to seafood priorities, which argues for monitoring by taxon and metal. Measuring tissue As speciation should come first, since the dietary ranking depends on it.

## 1. Introduction

Sediment-based indices are the standard entry point for heavy-metal assessment in semi-enclosed coastal bays. Metals delivered by rivers, mariculture, ports and urban runoff are readily retained by fine particles and organic matter, making surface sediments both a sink and a potential secondary source under changing redox and hydrodynamic conditions [1,2]. Risk is therefore commonly described by the geo-accumulation index, which quantifies enrichment relative to a regional background [3], and the potential ecological risk index (RI), which combines enrichment with metal-specific toxic-response factors [4]. In the Beibu Gulf of southern China, these sediment indices repeatedly identify Cd and secondarily Hg as priority metals [5,6,7,8]. In the Maowei Sea, a two-season sediment study likewise found Cd to contribute more than 80% of RI and linked its retention to nutrient enrichment, organic matter and seasonally changing hydrodynamics [2]. Following monitoring convention, the term heavy metals is used here for the seven regulated elements Cu, Pb, Zn, Cd, Cr, Hg and As, although As is strictly a metalloid.

The difficulty is that sediment risk is not the same object as seafood exposure. Tissue concentrations in aquatic animals reflect taxon-specific biodynamics, including dissolved uptake, dietary assimilation, storage, detoxification and efflux [9,10,11]. Bivalves often accumulate Cd and Zn efficiently, decapod crustaceans can carry high Cu burdens, and fish tend to show lower accumulation for many metals while retaining Hg in methylated and trophically transferred forms [9,12]. A metal can therefore dominate a sediment index because it is enriched and heavily weighted toxicologically yet fail to dominate the edible tissue or the final dietary hazard quotient. Whether sediment, bioaccumulation and dietary assessments identify the same priority metals is consequently an empirical question rather than a safe assumption.

Previous studies in Qinzhou Bay and the wider Beibu Gulf have examined parts of this chain. Gu et al. [13] combined sediment speciation with human-health implications for edible nekton in Qinzhou Bay, Qin et al. [14] assessed heavy metals and health risks in market bivalves from Beihai, Qinzhou and Fangchenggang, and Yang et al. [15] showed that metal burdens in farmed *Crassostrea hongkongensis* from Qinzhou Bay vary strongly with body size, season and growth conditions. These studies establish the regional relevance of seafood exposure, but they do not directly compare the metals prioritised by water and sediment assessment with those prioritised by taxon-specific accumulation and dietary intake in the same mariculture bay. That comparison matters because management decisions often move from environmental monitoring to seafood safety, even though the receptor changes from sediment-dwelling communities to the consumers of crustaceans, bivalves and fish; the metals of concern need not be the same at both ends.

The Maowei Sea provides a useful test case for this tiered comparison. It is a shallow semi-enclosed inner bay of Qinzhou Bay, covers about 135 km^2^, receives the Maoling, Dalan and Qin rivers, and experiences an irregular diurnal tide of 3.5 to 3.8 m [2]. Oyster rafts and cage culture occupy much of the inner basin, riverine nutrient loading has kept the bay moderately eutrophic [16], and the surrounding catchment includes urban, agricultural, port and petrochemical land uses [17]. In April 2022, one national-protocol survey commissioned for an environmental baseline assessment measured seven metals in water and surface sediment across the Maowei Sea and its outlet and collected seven commercially harvested taxa at twelve trawl stations within the bay for tissue analysis. All three tiers compared here are drawn from that survey record.

We examine whether the metals ranked highest by sediment indices are also the metals that dominate tissue burdens and dietary hazard, and therefore whether a sediment-derived priority list can be carried across to seafood safety. The accumulation literature makes it plausible that the ranking is taxon-conditional: the metal that leads in sediment need not lead in any taxon, because uptake and regulation differ between bivalves, crustaceans and fish, and because the dietary tier is weighted by toxicological reference values rather than by environmental enrichment. The comparison is made within a single survey and one laboratory, so that a divergence between the tiers cannot be attributed to differences between studies. The present analysis compares the 12-station arithmetic-mean tissue concentrations for each taxon and is a screening comparison rather than a statistical test of taxon differences. The objectives are to (i) evaluate contamination status and ecological risk for Cu, Pb, Zn, Cd, Cr, Hg and As in water and surface sediment, (ii) compare bioaccumulation factors and biota-sediment accumulation factors among crustaceans, bivalves and fish relative to the Maowei Sea environment where the animals were collected, and (iii) estimate deterministic and probabilistic dietary hazard for the seven sampled taxa across the inorganic-As fractions defined in Section 2.5.

## 2. Materials and Methods

### 2.1. Study Area and Sampling Design

**Figure 1:**
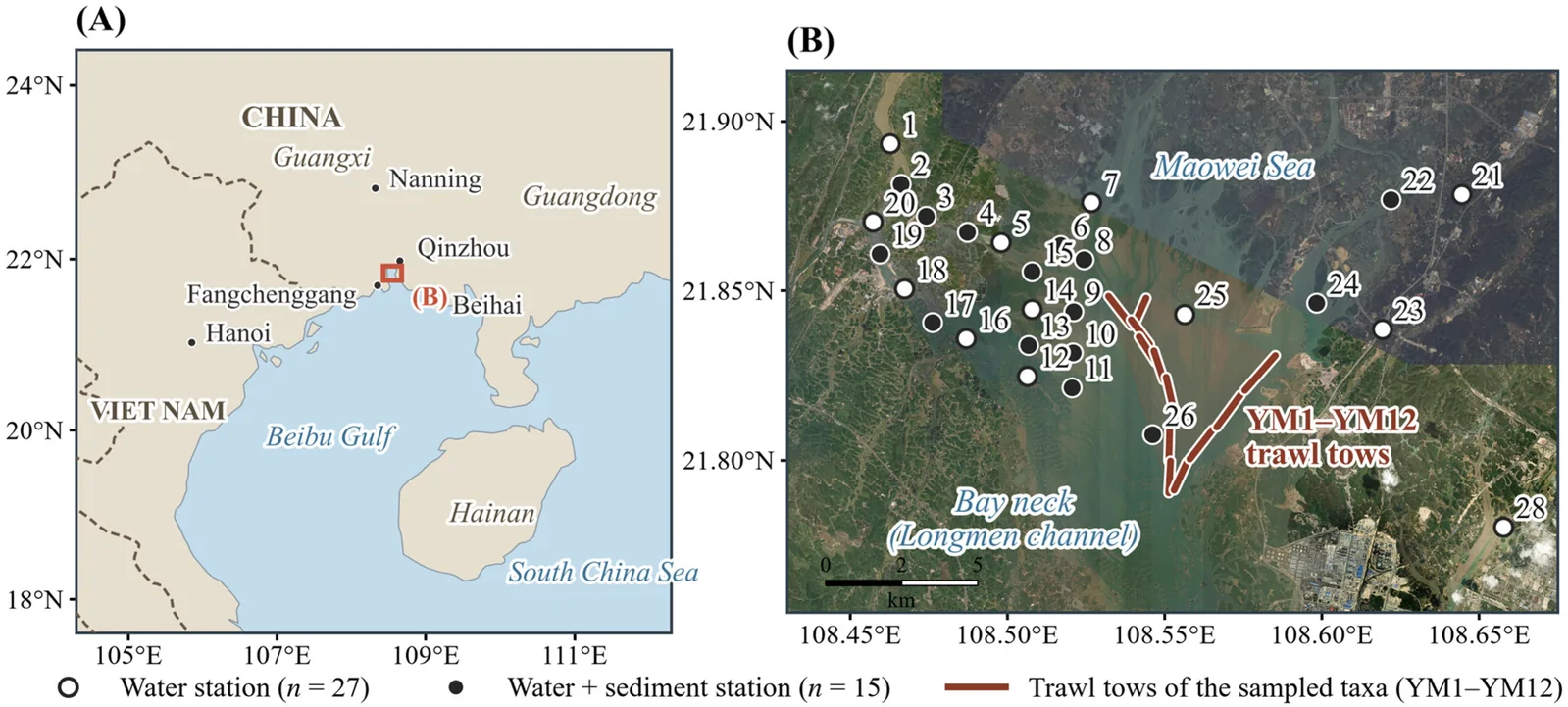
Sampling design in the Maowei Sea: 27 water stations and 15 sediment stations span the inner basin, the eastern river mouths and the outlet, with the twelve trawl stations of the sampled taxa spanning the central basin. (**A**) Study area on the northern coast of the Beibu Gulf; the red rectangle and red (B) mark the extent of panel (**B**); dashed lines are national boundaries. (**B**) Water-quality stations (open circles, water only; filled circles, water and surface sediment, *n* = 15); river-mouth stations are 21 and 22 in the inner basin and 23, 24 and 28 towards the outlet. The twelve tows (YM1 to YM12) at which the seven taxa were taken are drawn as lines, with their convex hull dotted; the stations within 3 km of a tow, used as the sensitivity denominator, are 6, 8 to 11, 14, 15 and 24 to 26. Basemap in (**B**): Esri World Imagery (Esri, Maxar, Earthstar Geographics and the GIS User Community); coastline and boundaries in (**A**): Natural Earth. The imagery is a composite acquired at an unknown tidal stage; the pale flats fringing the basin are intertidal and are exposed at low water.

Qinzhou Bay comprises the Maowei Sea, a narrow bay neck (the Longmen channel) and an outer bay open to the Beibu Gulf (Figure 1). The survey was carried out from 24 to 26 April 2022 by the Guangxi Academy of Sciences, with sampling, storage and transport following GB 17378.3-2007 [18] and biological sampling following GB/T 12763.6-2007 [19]. Of the 40 water-quality stations occupied across the bay, 27 lie within the Maowei Sea, the river mouths along its eastern margin and its outlet to the outer bay, and were used here; surface sediment was collected at 15 of these (Figure 1B). Salinity was measured in situ with a calibrated multi-parameter probe; surface values ranged from below 2 at the river-mouth stations 21 and 22 to 26.3, spanning the river-influenced inner basin to the tidal channel.

The biological survey was replicated in the field. Nekton were sampled at twelve bottom-trawl stations (YM1 to YM12) distributed across the Maowei Sea (108°32′ to 108°35′ E, 21°47′ to 21°51′ N), whose start and end positions span about 5.5 km east to west and 6.4 km north to south of the central basin (Figure 1B). Under GB/T 12763.6-2007 each tow constitutes a station: the catch of every tow was weighed and recorded separately, and the analysis sample was drawn from it at random. Each transect was towed for 20 to 60 min at about 4.5 km h^−1^ with a winged single-bag bottom trawl (4.0 m mouth width, 0.5 m mouth height, 8.0 m length, and 2.5 cm cod-end mesh); shooting and hauling positions were logged separately.

The tissue tier is resolved by station. Within each sampling station, tissue from individuals of the same taxon was pooled and homogenised, and the resulting station-level composites were analysed separately. For each taxon and metal, the reported concentration is the arithmetic mean of the 12 station-level results, with equal weight assigned to each station, and these means were used in the bioaccumulation and dietary-hazard calculations. The comparison between taxa is ordinal and descriptive; the consequences are set out in Section 3.6.

The spatial denominator is set to match where the animals were taken. The tissue samples of every taxon were taken in the Maowei Sea, across the area of the twelve trawl stations YM1 to YM12, so the bioaccumulation factors of Section 2.4 are computed against the mean water and sediment of the 27 water and 15 sediment stations that cover that area. Because the twelve trawl stations do not fill the bay uniformly, the factors were recomputed against the subset of stations lying within 3 km of a trawl transect, a buffer of about half the span of the trawled area (10 water stations 6, 8 to 11, 14, 15, 24 to 26; eight of these with sediment, 6, 8 to 11, 15, 24 and 26), and the two sets are compared in Supplementary Figure S1. The environmental tier itself (Section 2.3) is evaluated over all stations, because the bay-wide sediment ranking is what management applies to the bay as a whole and is the ranking whose transfer to the seafood tiers is under examination. The ranking over the near-trawl stations alone is reported alongside it in Section 3.1.

### 2.2. Sampling and Laboratory Analysis

Surface seawater was collected and preserved following GB 17378.3-2007 [18]. Samples for Cu, Pb, Zn, Cd, total Cr and As were filtered on board through 0.45 μm membranes into acid-cleaned 300 mL polyethylene bottles and acidified with HNO_3_ to pH < 2, so that the values of these six elements represent the dissolved fraction; samples for Hg were collected unfiltered in 250 mL brown glass bottles and acidified with H_2_SO_4_, so that Hg is reported as total Hg in the water column. Cu, Pb, Zn, Cd and Cr were preconcentrated by ammonium pyrrolidine dithiocarbamate and diethylammonium diethyldithiocarbamate chelation with solvent extraction. Cu, Pb, Cd and Cr were measured by graphite-furnace and Zn by flame atomic absorption spectrometry on an AA800 spectrometer (PerkinElmer, Waltham, MA, USA) with longitudinal Zeeman background correction, at the resonance lines of 324.8 nm (Cu), 283.3 nm (Pb), 228.8 nm (Cd), 357.9 nm (Cr) and 213.9 nm (Zn, air–acetylene flame). Hg and As were measured by atomic fluorescence spectrometry on an AFS-830 spectrometer (Beijing Jitian Instruments Co., Ltd., Beijing, China) with coded hollow-cathode lamps, argon as carrier and shield gas, 5% HCl as carrier solution and 2% KBH_4_ in 0.5% KOH as reductant, after sulfuric acid-persulfate digestion and borohydride reduction for Hg (cold vapour) and thiourea-ascorbic acid pre-reduction for As (hydride generation). Method detection limits were 0.20, 0.03, 3.10, 0.01, 0.40, 0.007 and 0.50 μg L^−1^ for Cu, Pb, Zn, Cd, Cr, Hg and As.

Surface sediment (0 to 2 cm) was recovered with a grab sampler and sealed in polyethylene bags. In the laboratory the samples were air-dried at room temperature, freed of shell fragments and debris, ground in an agate mortar and passed through a 160-mesh (about 0.1 mm) nylon sieve. Digestion followed GB 17378.5-2007 [18]: HNO_3_–HClO_4_–HF in closed PTFE vessels for Cu, Pb, Zn, Cd and Cr; HNO_3_–H_2_SO_4_ with V_2_O_5_ as catalyst for Hg; and aqua regia in a boiling water bath followed by thiourea-ascorbic acid pre-reduction for As. Cu and Zn were measured by flame and Pb, Cd and Cr by graphite-furnace atomic absorption on the AA800, and As by hydride-generation and Hg by cold-vapour atomic fluorescence on the AFS-830, under the instrument conditions given above. Detection limits were 0.5, 1.0, 6.0, 0.04, 2.0, 0.002 and 0.06 mg kg^−1^ dry weight for Cu, Pb, Zn, Cd, Cr, Hg and As. Organic carbon was determined by potassium dichromate oxidation and sulfide by methylene-blue spectrophotometry. All seven metals were detected in every sediment sample.

Biota were transported on ice in sealed boxes and analysed following GB 17378.6-2007 [18]. The prescribed tissue, muscle for fish and crustaceans and the whole soft body for bivalves, was dissected and rinsed with deionised water, and tissue from the same taxon was pooled and homogenised within each sampling station, with station-level composites kept separate for analysis. Triplicate sub-samples of a few grams fresh weight were taken from each station-level homogenate and digested with HNO_3_–HClO_4_ for Cu, Pb, Zn, Cd and Cr, with HNO_3_–H_2_SO_4_ and V_2_O_5_ for Hg, and with HNO_3_–HClO_4_ followed by thiourea-ascorbic acid pre-reduction for As. The digests were measured on the AA800 (Cu, Pb, Cd and Cr by graphite furnace, and Zn by flame) and the AFS-830 (Hg and As) as for the other matrices. Detection limits on a fresh-weight basis were 0.4, 0.04, 0.4, 0.005, 0.04, 0.002 and 0.2 mg kg^−1^ for Cu, Pb, Zn, Cd, Cr, Hg and As. Tissue concentrations are reported as 12-station arithmetic means on a fresh-weight basis. Each station-level composite comprised several individuals of similar, commercial size; the survey report gives neither their number nor the station-level concentrations, so station-level standard deviations cannot be derived from the summary means (Section 3.6).

The seven taxa were the shrimp *Metapenaeus joyneri* and the mitten crab *Eriocheir* sp. (crustaceans); the mangrove clam *Geloina erosa*, the oyster *Crassostrea* sp. and the hard clam *Meretrix meretrix* (bivalves); and the spotted sea bass *Lateolabrax maculatus* and the mullet *Chelon affinis* (fish). Species names follow the nekton and benthos inventories of the same survey, with two qualifications that are repeated in the note to the tissue-concentration table of Section 3.2. The oyster was collected in the same area as the trawl samples and is given at genus level because neither its culture status nor a species determination was recorded; *C. hongkongensis* is the dominant cultured and wild oyster of the bay. The mitten crab is recorded in the tissue table under its Chinese common name, conventionally rendered as *E. japonica*, whereas the survey’s separate nekton inventory gives the same entry the Latin name *E. sinensis*; as no voucher survives to check directly, it is retained at genus level in the text, tables and figures and tentatively associated with the Guangxi estuarine mitten-crab complex that includes *E. hepuensis*. Neither qualification affects the metal data, which are attached to the samples as analysed.

In the reported tissue results, Cr was below detection in all crustaceans and fish, Pb in all crustaceans and fish, and As in the shrimp and the sea bass. Non-detects were replaced by half the detection limit for the calculation of accumulation factors and hazard quotients and are flagged wherever they appear. The true values lie between zero and the detection limit, so the substituted values may be above or below them; they do not affect the identification of dominant metals except for As in the shrimp and the sea bass, which is treated explicitly in Section 3.3.

Analytical quality control followed GB 17378.2-2007 [18], which prescribes reagent blanks, certified reference materials of the corresponding matrix and parallel samples with every batch, and sets limits on spiked recoveries and on the relative deviation between parallel determinations. Each water and sediment concentration, and each station-level tissue result, is the mean of three parallel determinations rather than a single reading; the standard sets a duplicate requirement and the laboratory ran triplicates. Because the water-column Hg exceedance rests on a margin of about 24% between the mean concentration and the Class I limit, the sensitivity of the exceedance count to a hypothetical systematic bias is reported in Section 3.1.

### 2.3. Environmental Occurrence Indices

Water concentrations were benchmarked against Class I of the Chinese Sea Water Quality Standard GB 3097-1997 [20] (Cu 5, Pb 1, Zn 20, Cd 1, Cr 50, Hg 0.05 and As 20 μg L^−1^), and a water quality index was computed as the sum of the seven concentration-to-limit ratios, WQI = Σ Ci/Si. Sediment concentrations were benchmarked against Class I of the Marine Sediment Quality Standard GB 18668-2002 [21]. The geo-accumulation index was computed as I_geo_ = log_2_[C/(1.5 B)] [3], where B is the regional geochemical background of Guangxi coastal sediments [7]: Cu 15.8, Pb 28.9, Zn 75.8, Cd 0.09, Cr 39.9, Hg 0.029 and As 7.8 mg kg^−1^. Potential ecological risk was computed per metal as E_r_ = T_r_ × C/B, with toxic-response factors T_r_ of Zn 1, Cr 2, Cu 5, Pb 5, As 10, Cd 30 and Hg 40 [4], and summed to the risk index RI. Mean effects-range-median quotients (mERM-Q) were evaluated against the thresholds of Long and MacDonald [22,23]. Class boundaries are listed in Table 1. Index formulae and denominators are given in full in Supplementary Text S1, station-level data in Supplementary Table S1, and the sensitivity of BSAF to moisture content and of the hazard index to intake, body weight and As speciation in Supplementary Tables S2 and S3.

Acid-volatile sulfide and simultaneously extracted metals were not determined in the survey. Sediment bioavailability is therefore discussed qualitatively in relation to the measured total sulfide and organic carbon, and quantitatively only through the mERM-Q, which is an empirical toxicity benchmark rather than a speciation measure.

### 2.4. Bioaccumulation Factors

The field bioaccumulation factor relative to water was computed as BAF = C_biota_/C_water_, with C_biota_ representing the 12-station arithmetic-mean tissue concentration in mg kg^−1^ fresh weight and C_water_ in mg L^−1^, and the biota-sediment accumulation factor as BSAF = C_biota,dw_/C_sediment,dw_. The denominators are the mean concentrations of the 27 water and 15 sediment stations covering the Maowei Sea, the area in which the animals were taken (Section 2.1); the same factors computed against the stations within 3 km of a trawl transect are given in Supplementary Figure S1 as a sensitivity case. Because the animals were exposed through water, food and sediment at once and the water term is a zonal mean, the first ratio is a field bioaccumulation factor in the sense used by USEPA [24], not a laboratory bioconcentration factor. Fresh-weight tissue concentrations were converted to dry weight using literature moisture contents of 78% for crustacean muscle, 82% for bivalve soft tissue and 77% for fish muscle (Supplementary Table S2). BSAF values above 1 indicate tissue concentrations exceeding those of the sediment on a dry-weight basis; a BAF of 1000 is shown as a reference line, not as a regulatory classification. These factors are descriptive ratios used to rank metals within and across taxa rather than to estimate steady-state partitioning.

### 2.5. Dietary Hazard Assessment

The estimated daily intake of each metal was EDI = C × IR/BW, and the target hazard quotient THQ = (EF × ED × IR × C)/(RfD × BW × AT), following USEPA risk-assessment guidance [24,25]. Parameters were: exposure frequency EF = 365 d a^−1^, exposure duration ED = 70 a, averaging time AT = ED × 365 d, body weight BW = 60 kg and ingestion rate IR = 48.0 g d^−1^ (0.048 kg d^−1^ in the formula) for aquatic products, the national mean of the 2019 Chinese Food Consumption Survey [26]. Because the intake of any one taxon is unknown, each THQ is computed as if that taxon were the only seafood consumed, which is the conventional and conservative reading.

Oral reference doses for Zn (0.3), Cd (0.001), Cr (1.5, as Cr(III)), Hg (1 × 10^−4^, as methylmercury) and inorganic As (6 × 10^−5^ mg kg^−1^ d^−1^) were taken from the USEPA Integrated Risk Information System (IRIS). The As value is from the 2025 IRIS reassessment [27], which lowered the 1991 value of 3 × 10^−4^ five-fold; results with the superseded value are in Supplementary Table S3. IRIS provides no oral reference dose for Cu; the value of 0.04 mg kg^−1^ d^−1^ used here is the screening value of the USEPA Regional Screening Level tables, derived from the drinking-water action level for Cu [28,29], and the Cu quotient is read as a screening value. IRIS provides no reference dose for Pb either. The screening value of 0.0035 mg kg^−1^ d^−1^, derived from the provisional tolerable weekly intake of 25 μg kg^−1^ [30], withdrawn in 2010 because no threshold could be identified [31], is retained only so that Pb enters the sum; its quotient is read as a screening value.

The As quotient depends on the fraction of total As that is inorganic, which was not measured in this survey. Two assumed fractions are therefore carried side by side as scenarios, neither of which is a measurement for the taxa sampled here. The first, 10%, is a generic screening default widely applied in seafood hazard assessments. The second, 5%, is motivated by measured values in Chinese seafood: inorganic As was below 2% of total As in thirty types of Chinese seafood [32], 0.2 to 5.7% in eleven cultured shellfish species from Sanmen Bay [33], and arsenobetaine made up 81 to 99% of As compounds in molluscs and crustaceans of a South China estuary [34]. Fractions well above 10% have also been reported for bivalves at contaminated sites [35]. Because the local fraction is unknown, the dependence of every dietary result on the fraction is reported explicitly. The hazard index and the leading metal are also given for fractions of 2% and 1% (Supplementary Table S3), and for each taxon the fraction at which the hazard index reaches 1 and the fraction above which As overtakes the next metal are solved from the linear dependence of the As quotient on the fraction. Total Hg was used in place of methylmercury, a convention that overstates the Hg quotient in bivalves in particular. The hazard index HI is the sum of the seven THQ values; HI > 1 indicates a potential non-carcinogenic risk. Sensitivity to the intake assumptions was assessed for IR = 30, 48 and 100 g d^−1^ and BW = 60 and 70 kg, and the ingestion rate at which HI reaches 1 was solved for each taxon under both As fractions. Tissue concentrations were also compared with the maximum levels of the National Food Safety Standard GB 2762-2022 [36] for Pb, Cd, methylmercury (fish), inorganic As and Cr.

### 2.6. Probabilistic Exposure Assessment

Because the deterministic hazard quotients rest on point values for intake, body weight and speciation, a Monte Carlo simulation (20,000 iterations) was run to express the dietary hazard as a distribution. Ingestion rate was drawn from a lognormal distribution with geometric mean 40 g d^−1^ and geometric standard deviation 1.8, which reproduces the national mean of 48 g d^−1^ and places the 95th percentile near 105 g d^−1^. Body weight was drawn from a normal distribution with mean 60 kg and standard deviation 10 kg, truncated at 40 and 100 kg. Each tissue concentration was drawn from a normal distribution centred on the 12-station mean with a coefficient of variation of 10%, an assumed analytical variation that does not capture within-taxon biological variability; non-detects were drawn uniformly between zero and the detection limit. The inorganic fraction of As was drawn from a uniform distribution between 1 and 10%, spanning the two deterministic scenarios and the measured values in Chinese seafood elsewhere, and the methylmercury fraction of total Hg from uniform distributions of 70 to 95% for fish, 50 to 90% for crustaceans and 30 to 70% for bivalves. Reference doses were held fixed, and the same intake and body-weight draws were applied to every taxon so that taxa are compared for the same consumer. Because the As prior is an assumption, the whole simulation was repeated with an alternative prior of 0.2 to 5.7%, the range measured in the cultured shellfish of Sanmen Bay [33] (Supplementary Table S4).

The outputs are the median and 95th percentile of the hazard index, the modelled frequency with which it exceeds 1, the frequency with which any single quotient exceeds 1, and the frequency with which each metal leads the index. These frequencies describe parameter scenarios, not the fraction of real consumers at risk, because the model uses 12-station mean tissue concentrations without an empirical distribution of station-level concentrations, and the intake distribution is assumed rather than surveyed. Input sensitivity was summarised by Spearman rank correlation between each input draw and the hazard index.

All calculations were performed in Python 3.14.6 (Python Software Foundation, Wilmington, DE, USA) with NumPy 2.3.5, pandas 2.3.3 and SciPy 1.18.0; the scripts are available from the corresponding author.

## 3. Results and Discussion

### 3.1. Environmental Occurrence

**Figure 2:**
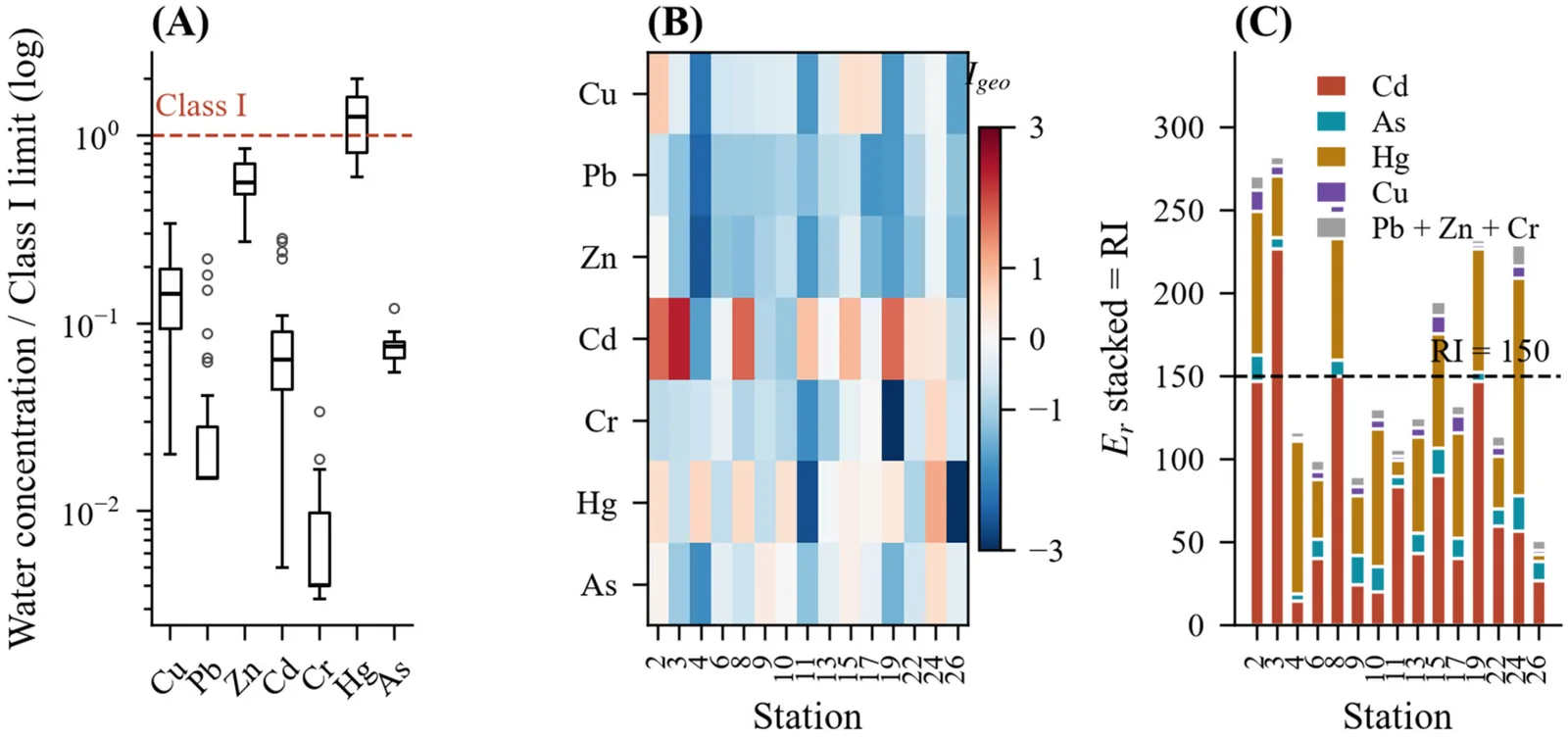
Hg is the only metal above the Class I seawater limit, and Cd and Hg together contributed 85% of the sediment ecological risk index. (**A**) Water-column concentrations at 27 stations as the ratio to the Class I limit of GB 3097-1997 (dashed line). (**B**) Geo-accumulation index of surface sediment at 15 stations relative to the Guangxi coastal background [7]; red is enrichment above 1.5 times background. (**C**) Ecological risk factors E_r_ stacked by metal to give RI at each station (Pb, Zn and Cr pooled); the dashed line marks the boundary between low and moderate risk.

Water-column metals were below the Class I standard at every station except for Hg (Figure 2A; Table 2), which exceeded the Class I limit at 16 of 27 stations, including 11 of the 14 stations of the inner basin. The water quality index averaged 2.19 (1.37 to 3.18) and was driven by the Hg term. Because the Hg values lie within a narrow band around the limit (0.030 to 0.10 μg L^−1^ against 0.05 μg L^−1^), the exceedance count was checked against an assumed multiplicative bias applied to every Hg value: a bias of −10% would leave the count at 16, −15% would reduce it from 16 to 14 stations, and +10% would raise it to 18. The check shows that the qualitative finding, Hg as the water-column metal most often above standard, survives a bias of this size. Concentrations of the other six metals were within the ranges reported for the northern Beibu Gulf over the past two decades [5,37], and the Hg exceedance is consistent with the position of Hg as the water-column metal most often above standard in this region [5].

In surface sediment (Table 2) the mean geo-accumulation index of Cu, Pb, Zn, Cr and As lay between −1.13 and −0.46, which is below 1.5 times the regional background at nearly all stations, and the Class I sediment limit was exceeded once each for Cu (station 2), Cr (station 24) and Cd (station 3). Cd and Hg were the exceptions (Figure 2B): Cd averaged 2.6 times the background, with I_geo_ above 0 at 8 of 15 stations and above 1 at four (stations 2, 3, 8 and 19), all in the inner basin, and Hg averaged 1.5 times the background, with I_geo_ above 0 at eight stations and above 1 at station 24 towards the outlet. Organic carbon ranged from 0.02 to 1.60% and sulfide from 0.74 to 18 mg kg^−1^, an 80-fold and a 24-fold spread respectively; organic carbon was highest at station 15 and sulfide at station 24, both of which also carried elevated Cd and Hg.

The ecological risk index followed the same two metals (Figure 2C). E_r_ for Cd averaged 78 (maximum 227) and reached the considerable-risk band (≥80) at six stations; E_r_ for Hg averaged 60 (maximum 131) and exceeded 80 at five. All other metals had E_r_ below 22 at every station. RI averaged 162 (51 to 283); nine stations fell in the low-risk band and six (stations 2, 3, 8, 15, 19 and 24) in the moderate band, none in the considerable band. Cd contributed 48% and Hg 37% of the summed RI, As 7% and Cu 4%, the remaining three metals less than 3% together. Restricted to the eight sediment stations within 3 km of a trawl transect (Section 2.1), the same two metals lead in the same order (Cd E_r_ 61, Hg 57; RI 51 to 259, mean 145), so the sediment ranking is the same for the bay as a whole and for the area the animals were taken from. The mERM-Q was low, 0.049 to 0.166 (mean 0.09), placing 12 stations in the lowest incidence class of Long and MacDonald [23] and three (2, 15 and 24) in the next.

These results agree with Huang et al. [2] in identifying Cd as the leading sediment risk factor in the Maowei Sea, with enrichment concentrated in the inner basin near the river mouths, and they extend that finding in one respect: Huang et al. [2] did not measure Hg, and in the present survey Hg accounted for more than a third of the risk index and was the only metal above standard in the water column. The Cd share of RI here (48%) is lower than the more than 80% they reported, largely because Hg absorbs part of the total; the absolute Cd concentrations are of the same order. The environmental tier characterises the Maowei Sea as a low-to-moderately contaminated bay in which Cd and Hg are the dominant risk metals, a description consistent with regional assessments over 2005 to 2020 [6,7].

### 3.2. Bioaccumulation in Crustaceans, Bivalves and Fish

**Figure 3:**
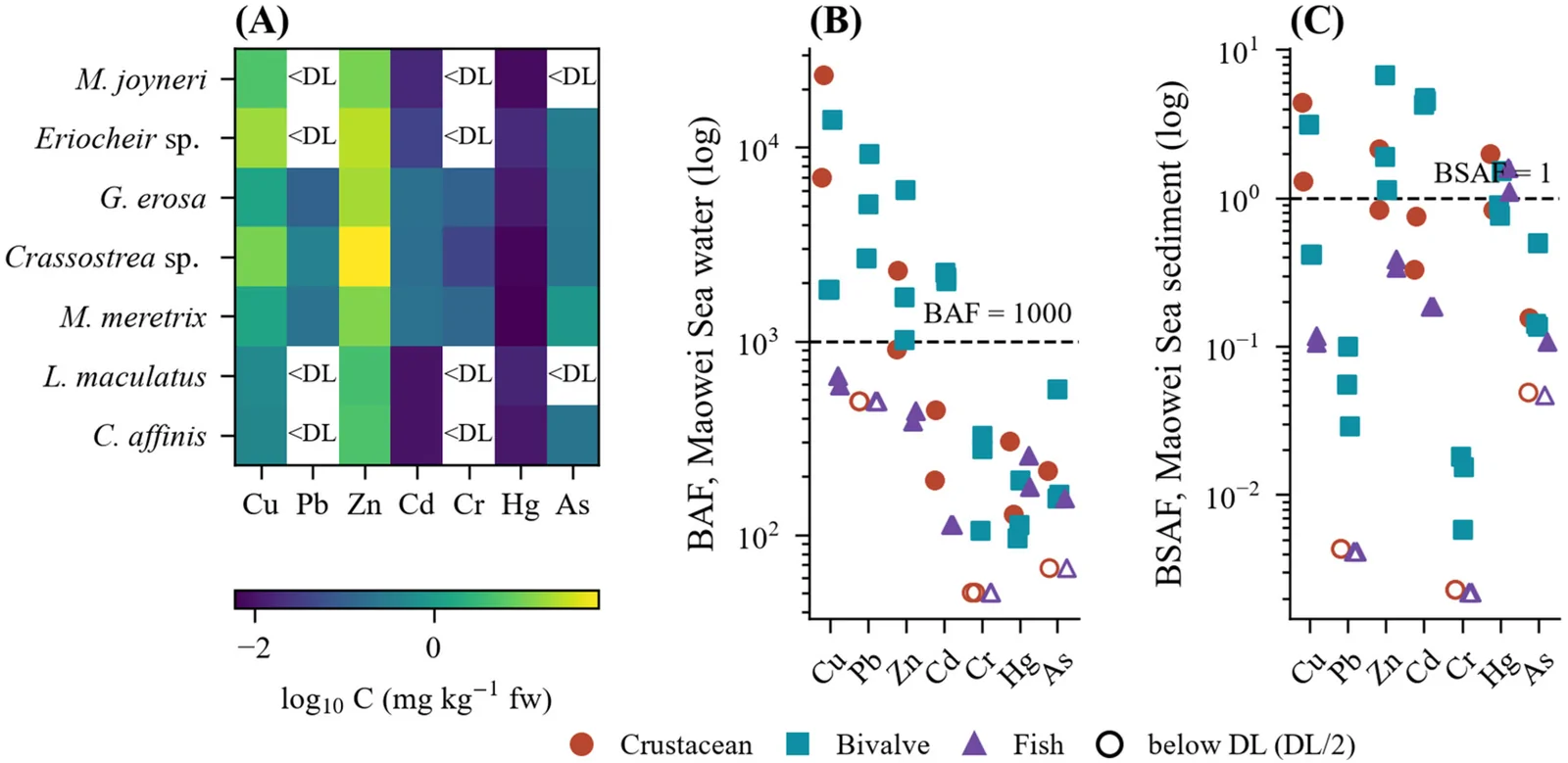
In the reported tissue data, bivalves accumulated Cd, crustaceans Cu, and fish only Hg above the Maowei Sea sediment level. (**A**) Tissue concentrations of the seven taxa (colour is log_10_ of the concentration in mg kg^−1^ fresh weight; <DL, below detection; values in Table 3). (**B**) Field bioaccumulation factors relative to the mean water concentration of the 27 Maowei Sea stations; the dashed line marks BAF = 1000 as a reference only. (**C**) Biota-sediment accumulation factors relative to the mean sediment concentration of the 15 Maowei Sea stations, dry-weight basis; the dashed line marks BSAF = 1. Symbol shape and colour identify the group (circles, crustaceans; squares, bivalves; triangles, fish); open symbols are computed from half the detection limit.

Tissue concentrations spanned three orders of magnitude across metals and differed widely across taxa for a single metal (Figure 3A; Table 3, block a). Zn was the most concentrated metal in every taxon; Cu was highest in the mitten crab and the oyster and lowest in the two fish; Cd concentrations were higher in the reported bivalve values than in the crustacean and fish values; Hg was highest in the crab and the two fish; Pb and Cr were detected only in bivalves; and As was highest in the hard clam and the crab. Two comparisons place these burdens in context. Converted to dry weight, the oyster fell below the reported range for Cd (about 1.0 against 7.9 to 72.2 mg kg^−1^) and near the lower end for Zn (about 390 against 282 to 17,003) and Cu (about 58 against 37 to 4012) of individually analysed farmed *C. hongkongensis* from Qinzhou Bay [15]; the hard clam Cd was within the range of market *M. meretrix* from the three Guangxi coastal cities [14]. Cr was below detection in every fish and crustacean, in contrast to the food-safety exceedance reported for Qinzhou Bay nekton by Gu et al. [13].

Expressed against the water and sediment of the Maowei Sea, the three groups ranked differently (Figure 3B,C; Table 3, blocks b and c). In the reported tissue data, the three bivalves carried the highest Cd factors (BSAF 4.3 to 4.8), about six-fold above the crab (0.76, itself below the sediment level) and more than twenty-fold above the fish. The oyster additionally exceeded the sediment level for Zn (6.78) and Cu (3.16) and carried the highest Pb BAF in the data set (9277), although its Pb BSAF remained at a tenth of the sediment level. The crustaceans were the accumulators of Cu, the crab holding the highest Cu factors of any taxon (BAF 23,691; BSAF 4.40) and the shrimp also exceeding the sediment level (1.30), whereas Cu in the fish stayed at about an eighth of it. The fish exceeded the sediment level for no metal except Hg (BSAF 1.60 and 1.10), which was also above it in the crab (1.98) and the mangrove clam (1.53) and just below it in the oyster (0.89). Pb and Cr stayed below a tenth of the sediment level in every taxon in which they were detected, and As did not reach half of it in any taxon. A similar taxon-conditional ordering is reported for Qinzhou Bay nekton [13].

This pattern is consistent with what comparative biodynamics would predict, and not simply with environmental abundance. Bivalves have limited excretion of Cd and store it bound to metallothionein-like proteins, which makes them efficient net accumulators under a wide range of ambient concentrations [9,11]. Oysters in particular accumulate Zn and Cu to concentrations that can reflect granule-storage physiology rather than local contamination alone [10], and Zn exposure itself enhances Cd and Cu accumulation in *C. hongkongensis* [38], consistent with the oyster being the only taxon here to accumulate all three. Decapods regulate Zn to a roughly constant body concentration but hold Cu in hemocyanin, so that Cu, an essential element that was not a leading environmental signal, has the highest BAF and BSAF of any metal in the crab. Fish regulate most metals across the gut and gill and retain principally methylmercury, which is assimilated with high efficiency from prey and eliminated slowly [12]. The BSAF ranking therefore indicates which metals in each taxon had tissue-to-environment ratios above 1, not which metal the sediment index ranks highest. In the reported tissue data, Cd, the sediment’s leading risk factor, exceeded the sediment level in the three bivalves alone; Hg, the second, in the fish, the crab and the mangrove clam; and Cu, although not prominent in the environmental indices, in the crustaceans and the oyster.

The factors are ratios of a 12-station mean tissue concentration to a zonal-mean environmental concentration; differences between taxa are reported as ordinal contrasts of a factor of two or more and not tested. The choice of environmental denominator has little effect. Recomputing the factors against the stations within 3 km of a trawl transect changes individual BSAF values by between 0.86- and 1.27-fold and individual BAF values by between 0.88- and 1.34-fold, and no metal crosses either reference line. The metal with the highest BSAF is the same in all seven taxa under both denominators; the highest BAF changes in one taxon, the mangrove clam, where Pb (2685) and Cd (2151) exchange places against the near-trawl water mean (2371 and 2420) (Supplementary Figure S1). Radii of 4 and 5 km lead to the same conclusions. The moisture-content assumption shifts all BSAF values of a taxon proportionally without altering the ranking within it, but it can move values near the reference line across it. With the moisture fraction five percentage points lower, the Hg BSAF of the mullet falls from 1.10 to 0.90; five points higher raises that of the oyster from 0.89 to 1.24 (Supplementary Table S2).

### 3.3. Dietary Hazard

No exceedance of the corresponding maximum levels in GB 2762-2022 was indicated under either speciation assumption (Table 3, block a). The highest ratios to the limit were Cd in the two clams (0.10 of the 2.0 mg kg^−1^ bivalve limit) and Pb in the oyster (0.25 of 1.5 mg kg^−1^). If all As were inorganic, As in the hard clam would reach 1.7 times the 0.5 mg kg^−1^ shellfish limit; at 10% and 5% the ratio falls to 0.17 and 0.08. Total Hg in both fish was less than 4% of the 0.5 mg kg^−1^ methylmercury limit. This food-standard comparison is therefore conditional on the assumed inorganic-As and methylmercury fractions, whereas the earlier regional dietary assessments similarly found no non-carcinogenic risk from Qinzhou Bay nekton [13] or from Guangxi market bivalves [14] under their own parameter choices.

**Figure 4:**
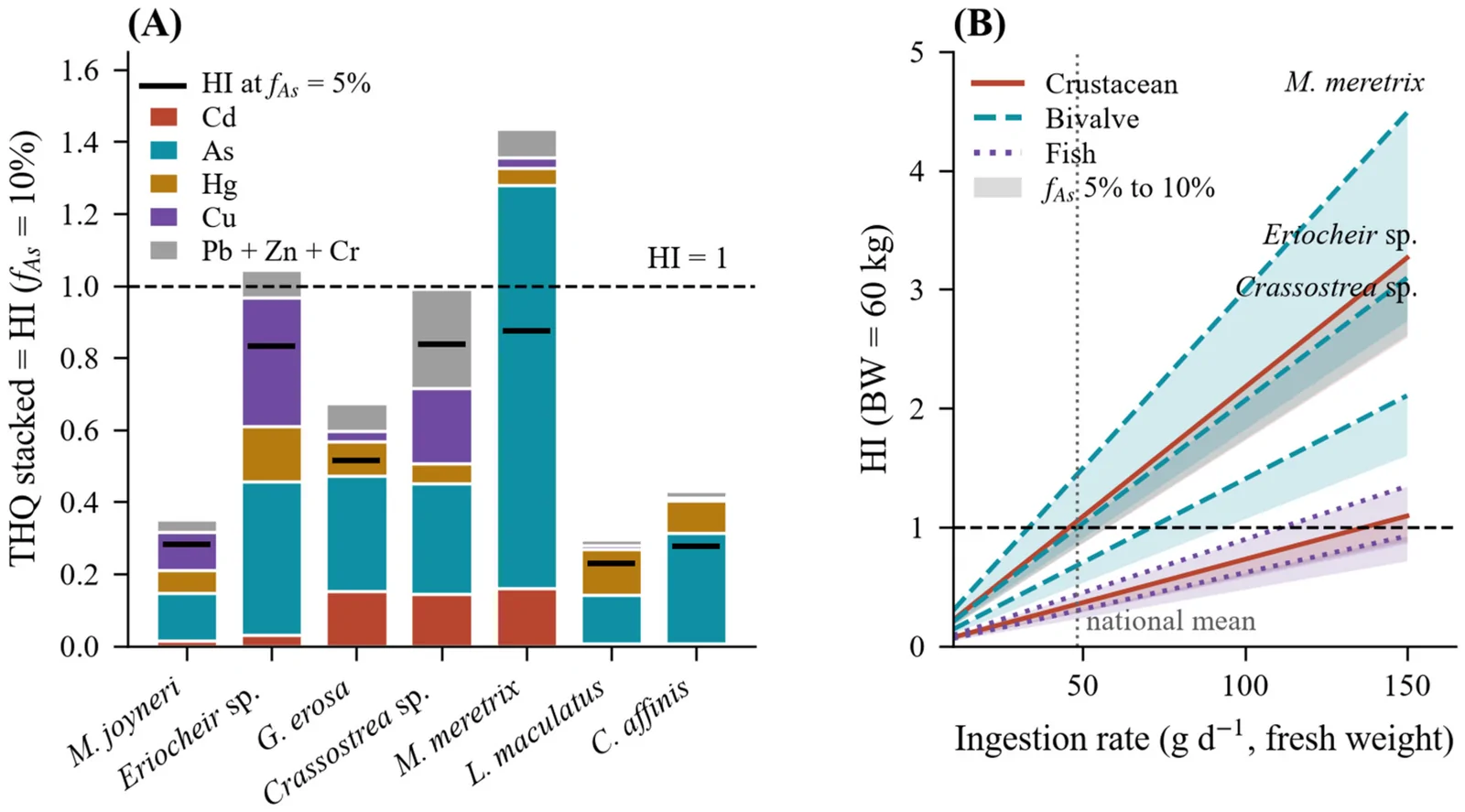
With the 2025 IRIS reference dose for inorganic As, the hard clam and the mitten crab exceed HI = 1 at the national-average intake only if 10% of their As is inorganic; at 5% no taxon exceeds the threshold, and the leading metal shifts from As to Cu in crustaceans and the oyster and to Hg in the sea bass. (**A**) Target hazard quotients stacked by metal to give the hazard index at an ingestion rate of 48 g d^−1^, body weight of 60 kg and an inorganic-As fraction of 10% (Pb, Zn and Cr pooled); black bars mark the hazard index at an inorganic-As fraction of 5%; the dashed line marks HI = 1. (**B**) Hazard index as a function of ingestion rate for each taxon at an inorganic-As fraction of 10% (lines) and the band down to the value at 5% (shading); colour and line style identify the group (solid, crustaceans; dashed, bivalves; dotted, fish), the three taxa that cross HI = 1 first are labelled, and the dotted vertical line marks the national-average ingestion rate.

The hazard index at the national-average ingestion rate spanned a factor of five across the seven taxa and its position relative to 1 depended on the As fraction (Figure 4A; Table 4). With inorganic As at 10% the hard clam (1.44) and the mitten crab (1.04) exceeded the threshold and the oyster approached it at 0.99; with inorganic As at 5% the same three taxa fell to 0.88, 0.83 and 0.84 and no taxon exceeded 1. The mangrove clam, the mullet, the shrimp and the sea bass stayed below 0.7 under both assumptions. These values also depend on the 2025 IRIS reference dose for inorganic As, which is five times lower than the 1991 value used in earlier assessments; with the superseded value every hazard index falls below 0.8 (Supplementary Table S3). The identity of the leading metal changed with the As fraction (Table 4). At 10%, As was the largest contributor wherever it was detected, from 31% of the index in the oyster to 78% in the hard clam, and its substituted quotient led even in the shrimp and the sea bass, where As was below detection. At 5%, Cu led in the shrimp (37%), the crab (43%) and the oyster (25%), As still led in the hard clam (64%) and the mullet (55%) and only narrowly in the mangrove clam (31% against 30% for Cd), and Hg led in the sea bass (56%). Because the As quotient is linear in the fraction, the crossover fraction above which As overtakes the next metal can be solved for each taxon: 1.4% in the hard clam, 2.9% in the mullet, 4.8% in the mangrove clam, 6.8% in the oyster, 8.0% in the shrimp, 8.4% in the crab and 9.6% in the sea bass (Supplementary Table S3). Below those fractions Cd leads in the two clams, Hg in the two fish and Cu in the crustaceans and the oyster. The hazard index reaches 1 at the national-average intake at a fraction of 6.1% for the hard clam, 9.0% for the crab and 10.3% for the oyster, and at no fraction below 20% for the other taxa. Behind the leading metal the composition of the index was consistent under both assumptions (Figure 4A): Cu and Hg in the crustaceans, Cd in the two clams, Cu, Zn and Cd in the oyster, and Hg in the fish. Earlier assessments of this region also gave As a large weight [13,14], but comparisons across studies should allow for differences in speciation assumptions and reference doses.

The hazard index is linear in the ingestion rate, so the margin to the threshold is best expressed as the intake at which each taxon reaches it (Figure 4B; Table 4 and Table S3). For a 60 kg consumer and inorganic As at 10% that intake is 33 g d^−1^ for the hard clam, 46 for the crab, 48 for the oyster, 71 for the mangrove clam, 112 for the mullet, 137 for the shrimp and 162 g d^−1^ for the sea bass. At 5% the corresponding intakes are 55, 58, 57, 93, 174, 170 and 209 g d^−1^. At 30 g d^−1^ no taxon exceeds 0.90 under either assumption; at 100 g d^−1^ the hard clam, the crab, the oyster and the mangrove clam exceed 1 at 10% (1.40 to 2.99) and the same four do so at 5% (1.07 to 1.82) (Supplementary Table S3). Raising body weight to 70 kg lowers every value by 14%.

Two assumptions govern the As result and one the Hg result. The inorganic fraction of total As decides whether the hard clam and the crab fall above or below the threshold at the national-average intake, and, between the 10% and 5% scenarios, which metal leads the index in four of the seven taxa (the shrimp, the crab, the oyster and the sea bass). The fraction was not measured, and the crossover fractions above are the quantity against which a future speciation measurement would be compared. The use of total Hg as methylmercury overstates the Hg quotient in bivalves, where the methylated fraction is typically well below that of fish; in fish the assumption is close to correct. The Monte Carlo analysis of the next section spans the As fraction from 1 to 10%, with an alternative prior of 0.2 to 5.7%, and the methylmercury fraction over group-specific ranges.

### 3.4. Probabilistic Dietary Hazard

**Figure 5:**
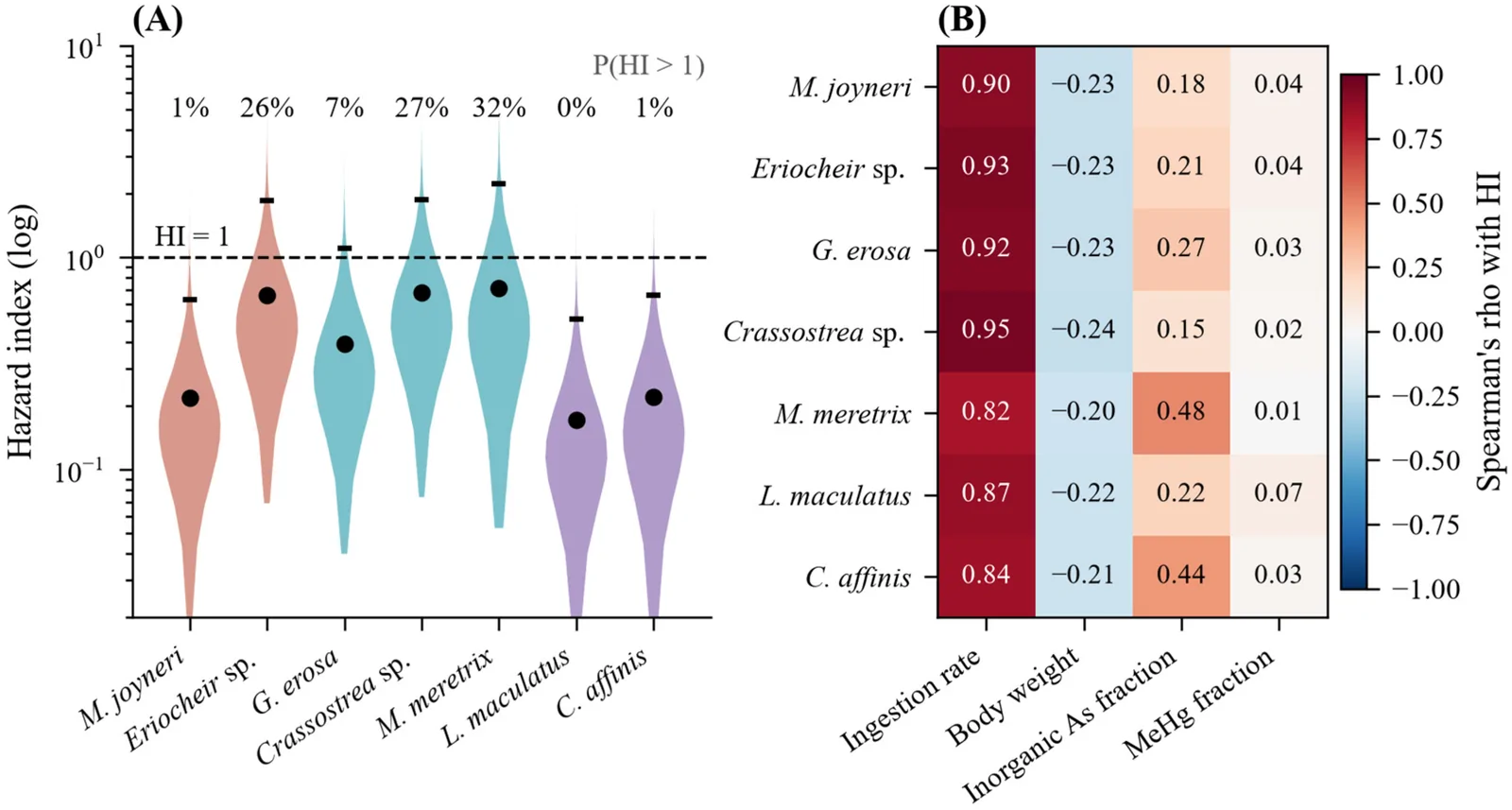
Under the assumed intake and speciation distributions the hard clam exceeds HI = 1 in 32% of scenarios and the oyster and the crab in about 26%, and the inorganic As fraction is the input that matters most after ingestion rate in the taxa whose index is led by As. (**A**) Monte Carlo distributions of the hazard index for each taxon (20,000 iterations; dots, medians; dashes, 95th percentiles; the dashed line marks HI = 1, and the percentages give the modelled exceedance frequency under the assumed distributions). (**B**) Spearman rank correlation between each uncertain input and the hazard index.

Every median hazard index fell below 1, from 0.17 in the sea bass to 0.71 in the hard clam, because the intake distribution is right-skewed and the As fraction was drawn from 1 to 10% rather than fixed at 10% (Figure 5A; Table 5). The 95th percentiles reached 2.23 for the hard clam and about 1.9 for the oyster and the crab. The modelled frequency with which the index exceeded 1 under the assumed input distributions was 32% for the hard clam, 27% for the oyster, 26% for the crab, 7.1% for the mangrove clam and 1.2% or less for the other three taxa (Table 5). Only in the hard clam did a single quotient, that of As, exceed 1 in an appreciable share of iterations (17%); in the crab and the oyster the exceedances arose from the sum of several quotients. The leading metal was taxon-specific rather than uniformly As: Cu led in 74% of iterations for the shrimp, 82% for the crab and 54% for the oyster, As in 95% for the hard clam, 84% for the mullet and 58% for the mangrove clam (Cd in the remaining 42%), and Hg in 74% for the sea bass. These frequencies are properties of the assumed As prior. Under the alternative prior of 0.2 to 5.7% (Supplementary Table S4) the exceedance frequency of the hard clam falls to 15%, the oyster and the crab to 22% and 19%, and every other taxon stays below 4%. Cu then leads in the crustaceans and the oyster in 79 to 100% of iterations, Cd in the mangrove clam in 83%, As in the hard clam in 78% and in the mullet in 60%, and Hg in the sea bass in 94%.

Ingestion rate remained the dominant input (Figure 5B), with a rank correlation of 0.82 to 0.95 with the hazard index in every taxon, against −0.20 to −0.24 for body weight. The inorganic As fraction ranked second only in the taxa whose index is led by As, the hard clam (0.48), the mullet (0.44) and the mangrove clam (0.27); in the other four taxa its correlation (0.15 to 0.22) was smaller in magnitude than that of body weight. The methylmercury fraction never exceeded 0.07. The practical reading is that consumption advice for this bay hinges on two quantities, how much of a taxon is eaten and, for the clams and the mullet, what fraction of its As is inorganic, and that the second is the measurement most worth adding to future surveys.

### 3.5. Do the Three Tiers Agree?

**Figure 6:**
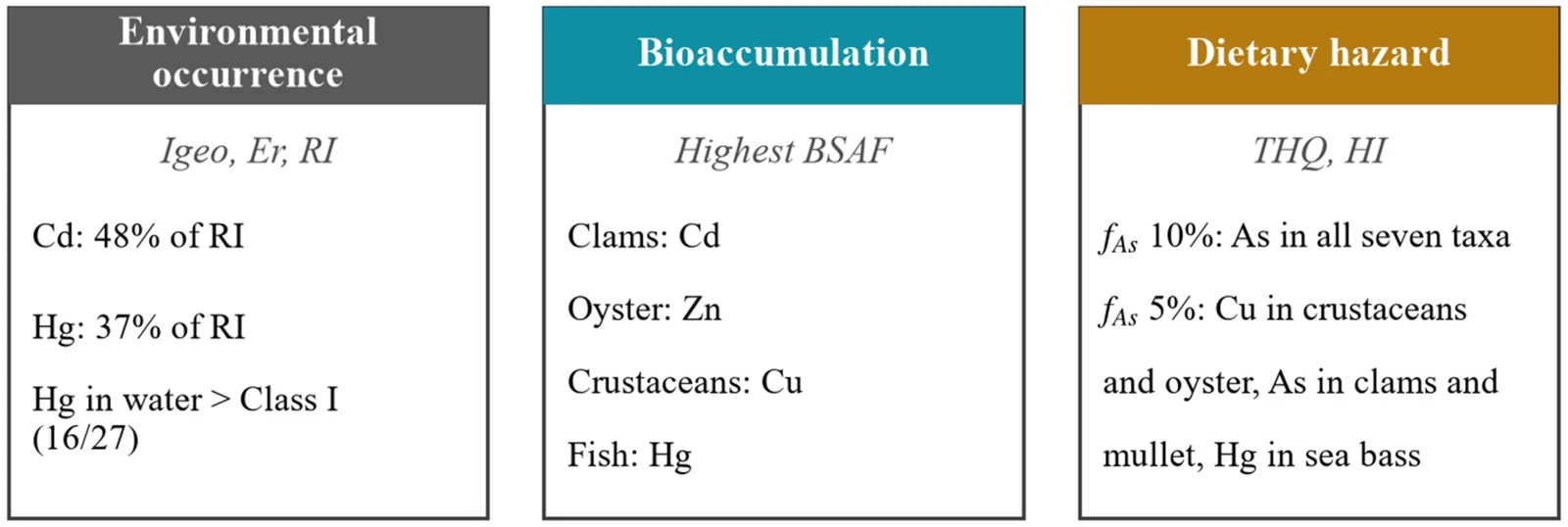
Comparison of the assessment tiers for the seven sampled taxa. The environmental panel shows Cd and Hg contributions to the sediment risk index and the frequency of water-column Hg exceeding Class I. The bioaccumulation panel shows the highest BSAF for each taxon, using whole-bay sediment denominators: Cd in the two clams, Zn in the oyster, Cu in the crustaceans and Hg in the fish. The dietary panel shows the leading THQ under the 10% and 5% inorganic-As scenarios. Header colours identify the three assessment tiers (grey, environmental occurrence; teal, bioaccumulation; orange, dietary hazard).

The three tiers rank metals on different quantities, and the comparison below is between those rankings rather than between a single measure applied three times as follows: the environmental tier uses the per-metal ecological risk factor E_r_, the bioaccumulation tier the highest BSAF for the synthesis in Figure 6 (with BAF reported separately), and the dietary tier the target hazard quotient. The distinction matters for the middle tier in particular, because the metal present at the highest tissue concentration is not the metal with the highest enrichment relative to the environment. Zn was the most concentrated metal in every taxon (Table 3, block a), whereas the taxon-specific leading metals identified below are those of the accumulation factors (Table 3, blocks b and c).

Set side by side, the three tiers identify different metals (Figure 6). The environmental tier flags Cd and Hg, both in the bay as a whole and over the near-trawl stations alone. The highest BSAF is for Cd in the two clams, Zn in the oyster, Cu in the two crustaceans and Hg in the two fish. The dietary tier ranks As first in every taxon if 10% of the As is inorganic; at 5% it ranks Cu first in the crustaceans and the oyster, As first in the two clams and the mullet, and Hg first in the sea bass. How far the dietary tier departs from the sediment ranking depends on the unmeasured As fraction. The metal leading the dietary tier is not one of the two leading sediment risk drivers in all seven taxa at 10%, six at 5% (the sea bass, led by Hg, is the exception), four at 2% and three at 1%, because at low fractions Cd takes over in the clams and Hg in both fish (Supplementary Table S3). With other model inputs fixed, the departure persists across the As fractions considered for the two crustaceans and the oyster, in which Cu, absent from the top of the sediment ranking, leads at every fraction below its own crossover (6.8% in the oyster, 8.0% in the shrimp and 8.4% in the crab) and As above it. Neither As nor Cu has a positive mean geo-accumulation index or ranks among the leading contributors to the sediment risk index, yet together they contribute two thirds to three quarters of the crab’s hazard index. The discrepancy is consistent with each tier weighting the metals differently. The sediment index weights by toxic-response factor and by enrichment over background, which favours Cd (T_r_ = 30) and Hg (T_r_ = 40). Bioaccumulation weights by taxon-specific uptake and retention, which is consistent with the different BSAF leaders in the clams, oyster, crustaceans and fish. The hazard quotient weights by the ratio of tissue concentration to reference dose, favouring metals with low reference doses (As after its 2025 revision, Hg, Cd) or high tissue concentrations (Cu in crustaceans, Zn and Cu in the oyster); the balance depends on the speciation assumption.

Two observations follow for a mariculture bay such as this, both at the screening level of the present design. The first is that sediment indices remain a useful environmental screening tier: they identify Cd and Hg as the metals driving potential ecological risk in the Maowei Sea. The second is that the leading dietary metal differed from the two sediment risk leaders for the crustaceans and the oyster under any As fraction considered, and for the clams and the mullet under the two main scenarios. A monitoring programme that inferred the metals to watch in oysters from the sediment index would watch Cd and would capture one important bivalve signal. One that inferred the metals to watch in crabs would watch Cd and Hg and would miss Cu and As, which together carry two thirds to three quarters of the hazard index. These screening results suggest that seafood monitoring in this bay would benefit from a taxon-and-metal design. For the Maowei Sea the candidate combinations for replicated monitoring are As and Cd in bivalves, Cu, As and Hg in crustaceans and Hg and As in fish, with tissue As speciation as the first measurement to add; the intake-at-threshold values reported here offer a direct way to frame consumption advice for high-intake groups once those measurements exist.

A comparison with the previous assessment of the bay is instructive. Huang et al. [2] concluded from sediment alone that Cd should be prioritised in the monitoring and management of the Maowei Sea. The sediment results here support that conclusion for the environmental tier, but the reported tissue data suggest that the priority is taxon-conditional. Cd is a defensible priority for the oyster and the clams, which are the bay’s principal product; in the two fish it stayed at a third of the sediment level and contributed less than 3% of the hazard index, in the crustaceans it was of secondary importance, and at the dietary tier it was overtaken by As or Cu in every taxon under both scenarios. The two studies, three years apart and with different metal suites, converge on the sediment result while the present study adds a divergent biological result; the divergence is consistent with receptor-specific accumulation rather than with environmental abundance alone.

### 3.6. Limitations

Four features of the design bound the inference drawn here. First, the survey covers one season, April 2022, at the transition into the wet season; Huang et al. [2] showed that sediment Cd in this bay varies between wet and dry seasons, and tissue concentrations in bivalves are known to vary with reproductive condition, so the absolute values here should not be read as annual means. The comparison rests on which metal leads each tier rather than on these absolute values; at the bioaccumulation tier that ordering follows taxon-specific uptake and storage [9,11] and is similar to the ordering reported for Qinzhou Bay nekton in an earlier survey [13]. Second, each taxon is represented by the mean of 12 separately analysed station-level composites, but the report gives only the means, so station-level variance and statistical differences between taxa cannot be estimated and all contrasts are ordinal; pooling within each station also averages over the individuals caught there. Third, the twelve trawl stations span the central basin but do not reach the innermost river mouths, where sediment Cd is highest, so the factors describe animals of the central basin. The denominators are area means rather than the exposure of each animal at its own station; the near-trawl sensitivity case preserves the leading BSAF metal for every taxon and the reference-line classifications but changes the leading BAF metal of the mangrove clam from Pb to Cd (Section 3.2).

Fourth, neither acid-volatile sulfide nor metal speciation in tissue (methylmercury, inorganic As) was measured, so the bioavailability step between sediment and biota is inferred from the accumulation factors rather than observed. The Hg and As hazard quotients therefore rest on assumed speciation fractions, on which the dietary conclusions are conditional (Section 3.3). A repeat survey that reports the station-level tissue values in both seasons, with Hg and As speciation in tissue, would allow the taxon-conditional pattern reported here to be tested statistically and resolved by station.

## 4. Conclusions

Water, surface sediment and 12-station mean tissue concentrations of seven sampled taxa from the Maowei Sea were compared in one survey to examine whether environmental occurrence, bioaccumulation and dietary hazard identify the same priority metals. In sediment, Cd and Hg together contributed 85% of an ecological risk index that was low to moderate at all 15 stations; water-column Hg exceeded the Class I seawater limit at 16 of 27 stations. Bioaccumulation against the Maowei Sea environment was group-specific: Cd BSAF exceeded 1 in all three bivalves, the oyster also exceeded the sediment level for Zn and Cu and carried the highest Pb BAF, the *Eriocheir* crab held the highest Cu factors, and the fish exceeded the sediment level for Hg alone. No food-limit exceedance was indicated under the speciation fractions assumed here. With the 2025 IRIS reference dose for inorganic As the hazard index at the national-average intake exceeded 1 for the hard clam (1.44) and the mitten crab (1.04) if 10% of the As is inorganic and fell to 0.88 and 0.83 if 5% is. As led the index in every taxon at 10%; at 5% Cu led in the crustaceans and the oyster, As in the clams and the mullet and Hg in the sea bass. Monte Carlo simulation gave modelled exceedance frequencies of 32%, 27% and 26% for the hard clam, the oyster and the crab under an As prior of 1 to 10%, and 15%, 22% and 19% under a prior of 0.2 to 5.7%. The three tiers thus disagree on which metal matters, consistent with receptor-specific accumulation and with the different weightings of the three index families. The extent of the disagreement depends on the unmeasured inorganic-As fraction: the leading dietary metal differed from the two leading sediment risk drivers in all seven taxa at 10%, six at 5% and three at 1%; for the crustaceans and the oyster the departure persists across the fractions considered, with other model inputs fixed. Sediment indices remain useful for environmental screening, but in this single-survey comparison they did not identify the metals that led the dietary tier in most taxa under the two main scenarios. These screening results suggest candidate taxon–metal combinations for replicated monitoring: As and Cd in bivalves, Cu, As and Hg in crustaceans and Hg and As in fish, with tissue As speciation as the first measurement to add.

## Figures and Tables

**Table 1:** Indices, parameters and class boundaries used in the three tiers of assessment.

Tier	Index	Formula	Parameters	Class Boundaries
Environment	I_geo_	log_2_[C/(1.5 B)]	B from Bi et al. [7]	≤0 unpolluted; 0–1 unpolluted to moderate; 1–2 moderate; 2–3 moderate to heavy
Environment	E_r_, RI	E_r_ = T_r_ C/B; RI = ΣE_r_	T_r_: Zn 1, Cr 2, Cu 5, Pb 5, As 10, Cd 30, Hg 40	E_r_ < 40 low, 40–80 moderate, 80–160 considerable; RI < 150 low, 150–300 moderate, 300–600 considerable
Environment	mERM-Q	mean(C/ERM)	ERM from Long et al. [22]	<0.1: 9% incidence of toxicity; 0.1–0.5: 21%
Bioaccumulation	BAF (field, relative to water)	C_biota,fw_/C_water_	Mean water of the 27 Maowei Sea stations (near-trawl subset as sensitivity)	1000 shown as a reference line, not a regulatory classification for metals
Bioaccumulation	BSAF	C_biota,dw_/C_sed,dw_	Moisture 78/82/77% (crustacean/bivalve/fish); mean sediment of the 15 Maowei Sea stations (near-trawl subset as sensitivity)	>1 tissue exceeds sediment
Dietary	THQ, HI	(EF ED IR C)/(RfD BW AT); HI = ΣTHQ	EF 365, ED 70, IR 48.0 g d^−1^, BW 60 kg; RfD (mg kg^−1^ d^−1^): Zn 0.3, Cd 0.001, Cr 1.5, Hg 1 × 10^−4^, inorganic As 6 × 10^−5^ (IRIS; As 2025 value, 3 × 10^−4^ before 2025); Cu 0.04 (USEPA RSL screening value; no IRIS RfD); Pb 0.0035 screening value (JECFA 1993 PTWI, withdrawn 2010; no IRIS RfD); inorganic As = 10% and 5% of total (2% and 1% in Table S3)	THQ or HI > 1: potential non-carcinogenic risk

**Table 2:** Metal concentrations in surface water (*n* = 27) and surface sediment (*n* = 15) of the Maowei Sea and its outlet, April 2022, with regulatory limits and regional background.

Metal	Water Mean (Range), μg L^−1^	Class I Limit, μg L^−1^	Stations > Class I	Sediment Mean (Range), mg kg^−1^ dw	Class I Limit, mg kg^−1^	Background [7]	I_geo_ Mean (Max)	E_r_ Mean (Max)
Cu	0.76 (0.10–1.70)	5	0	18.5 (5.3–40.6)	35	15.8	−0.59 (0.78)	5.8 (12.8)
Pb	0.041 (0.015–0.22)	1	0	21.1 (8.4–39.9)	60	28.9	−1.13 (−0.12)	3.6 (6.9)
Zn	11.6 (5.4–16.9)	20	0	57.5 (19.1–114)	150	75.8	−1.12 (0.00)	0.8 (1.5)
Cd	0.088 (0.005–0.28)	1	0	0.234 (0.044–0.68)	0.5	0.09	0.35 (2.33)	78.0 (227)
Cr	0.40 (0.17–1.70)	50	0	39.8 (7.3–92.3)	80	39.9	−0.77 (0.63)	2.0 (4.6)
Hg	0.062 (0.030–0.10)	0.05	16	0.044 (0.003–0.095)	0.2	0.029	−0.38 (1.13)	60.0 (131)
As	1.49 (1.10–2.40)	20	0	9.3 (3.2–16.9)	20	7.8	−0.46 (0.53)	11.9 (21.7)

**Table 3:** Arithmetic-mean metal concentrations in the seven sampled taxa (12 station-level composites per taxon; block a, mg kg^−1^ fresh weight, with the GB 2762-2022 maximum levels for each group), field bioaccumulation factors relative to the mean water concentration of the 27 Maowei Sea stations (block b, BAF) and biota-sediment accumulation factors relative to the mean sediment concentration of the 15 Maowei Sea stations on a dry-weight basis (block c, BSAF). “<DL” is below the detection limit; factors in parentheses are computed from half the detection limit and are substituted estimates. Taxonomic note: the mitten crab is given as *Eriocheir* sp. because the survey’s tissue table and nekton inventory assign the same sample to different species and no voucher survives; the oyster is given as *Crassostrea* sp. because neither a species determination nor its culture status was recorded (*C. hongkongensis* is the dominant cultured and wild oyster of the bay). Standard deviations are not given because the underlying station-level values are not available in the report.

Block	Taxon (Group)	Cu	Pb	Zn	Cd	Cr	Hg	As
(a) Concentration	*M. joyneri* (Crustacean)	5.3	<DL	10.6	0.017	<DL	0.008	<DL
(a) Concentration	*Eriocheir* sp. (Crustacean)	17.9	<DL	27.1	0.039	<DL	0.019	0.32
(a) Concentration	*G. erosa* (Bivalve)	1.4	0.11	19.7	0.19	0.11	0.012	0.24
(a) Concentration	*Crassostrea* sp. (Bivalve)	10.5	0.38	70.2	0.18	0.042	0.007	0.23
(a) Concentration	*M. meretrix* (Bivalve)	1.4	0.21	11.8	0.2	0.13	0.006	0.84
(a) Concentration	*L. maculatus* (Fish)	0.5	<DL	4.5	0.01	<DL	0.016	<DL
(a) Concentration	*C. affinis* (Fish)	0.45	<DL	5.1	0.01	<DL	0.011	0.23
(a) GB 2762-2022 limit	Fish	–	0.5	–	0.1	2.0	0.5 (MeHg)	0.1 (inorganic)
(a) GB 2762-2022 limit	Crustaceans	–	0.5	–	0.5	2.0	0.5	0.5 (inorganic)
(a) GB 2762-2022 limit	Bivalves	–	1.5	–	2.0	2.0	0.5	0.5 (inorganic)
(b) BAF	*M. joyneri*	7015	(488)	911	192	(50)	128	(67)
(b) BAF	*Eriocheir* sp.	23,691	(488)	2330	442	(50)	305	215
(b) BAF	*G. erosa*	1853	2685	1693	2151	277	193	162
(b) BAF	*Crassostrea* sp.	13,897	9277	6034	2038	106	112	155
(b) BAF	*M. meretrix*	1853	5127	1014	2264	327	96	566
(b) BAF	*L. maculatus*	662	(488)	387	113	(50)	257	(67)
(b) BAF	*C. affinis*	596	(488)	438	113	(50)	177	155
(c) BSAF	*M. joyneri*	1.30	(0.004)	0.84	0.33	(0.002)	0.84	(0.05)
(c) BSAF	*Eriocheir* sp.	4.40	(0.004)	2.14	0.76	(0.002)	1.98	0.16
(c) BSAF	*G. erosa*	0.42	0.03	1.90	4.51	0.02	1.53	0.14
(c) BSAF	*Crassostrea* sp.	3.16	0.10	6.78	4.28	0.006	0.89	0.14
(c) BSAF	*M. meretrix*	0.42	0.06	1.14	4.75	0.02	0.77	0.50
(c) BSAF	*L. maculatus*	0.12	(0.004)	0.34	0.19	(0.002)	1.60	(0.05)
(c) BSAF	*C. affinis*	0.11	(0.004)	0.39	0.19	(0.002)	1.10	0.11

**Table 4:** Target hazard quotients at an ingestion rate of 48.0 g d^−1^, body weight of 60 kg and an inorganic-As fraction of 10%, with the 2025 IRIS reference dose for inorganic As; the hazard index at inorganic-As fractions of 10% and 5%; the leading metal and its share of the index under each fraction; and the ingestion rate at which HI = 1 under each fraction. The As quotient of the shrimp and the sea bass is computed from half the detection limit and is a substituted estimate. Results with the superseded 1991 As reference dose are given in Supplementary Table S3.

Taxon	Cu	Zn	Cd	Pb	Cr	Hg	As (10%)	HI (10%)	HI (5%)	Leading Metal (Share), 10% → 5%	IR at HI = 1, 10%/5% (g d^−1^)
*M. joyneri*	0.106	0.028	0.014	0.005	<0.001	0.064	(0.133)	0.35	0.28	As (38%) → Cu (37%)	137/170
*Eriocheir* sp.	0.358	0.072	0.031	0.005	<0.001	0.152	0.427	1.04	0.83	As (41%) → Cu (43%)	46/58
*G. erosa*	0.028	0.053	0.152	0.025	<0.001	0.096	0.320	0.67	0.51	As (47%) → As (31%)	71/93
*Crassostrea* sp.	0.210	0.187	0.144	0.087	<0.001	0.056	0.307	0.99	0.84	As (31%) → Cu (25%)	48/57
*M. meretrix*	0.028	0.031	0.160	0.048	<0.001	0.048	1.120	1.44	0.88	As (78%) → As (64%)	33/55
*L. maculatus*	0.010	0.012	0.008	0.005	<0.001	0.128	(0.133)	0.30	0.23	As (45%) → Hg (56%)	162/209
*C. affinis*	0.009	0.014	0.008	0.005	<0.001	0.088	0.307	0.43	0.28	As (71%) → As (55%)	112/174

**Table 5:** Monte Carlo dietary-hazard summary (20,000 iterations; inorganic-As fraction uniform between 1 and 10%; the alternative prior of 0.2 to 5.7% is in Supplementary Table S4): median and 95th percentile of the hazard index, the modelled frequency with which HI exceeds 1 and with which any single THQ exceeds 1 under the assumed input distributions, the metal that most often leads the index (frequency of leading; median share of HI in the iterations in which it leads), and the frequency with which As leads.

Taxon	HI Median	HI 95th Percentile	Frequency HI > 1	Frequency Any THQ > 1	Most Frequent Leading Metal (Frequency; Median Share When Leading)	Frequency as Leads
*M. joyneri*	0.22	0.63	0.9%	0.0%	Cu (74%; 44%)	26%
*Eriocheir* sp.	0.66	1.85	25.7%	2.7%	Cu (82%; 46%)	18%
*G. erosa*	0.39	1.11	7.1%	0.3%	As (58%; 43%)	58%
*Crassostrea* sp.	0.68	1.88	26.9%	0.4%	Cu (54%; 28%)	33%
*M. meretrix*	0.71	2.23	31.7%	16.6%	As (95%; 68%)	95%
*L. maculatus*	0.17	0.51	0.4%	0.0%	Hg (74%; 59%)	26%
*C. affinis*	0.22	0.66	1.2%	0.2%	As (84%; 63%)	84%

## Data Availability

Station-level water and sediment data are provided in Supplementary Table S1, and the 12-station arithmetic-mean tissue concentrations in Table 3. The analysis scripts are available from the corresponding author on reasonable request.

## Supplementary Materials

The following supporting information can be downloaded at https://www.mdpi.com/article/10.3390/toxics14100875/s1, Text S1: Index formulae, denominators and class boundaries; Table S1: Station-level data; Table S2: Sensitivity of the biota-sediment accumulation factor to the assumed moisture content; Table S3: Sensitivity of the hazard index to ingestion rate, body weight, the inorganic-As fraction and the As reference dose; Table S4: Monte Carlo summary under the alternative inorganic-As prior; Figure S1: Whole-bay versus near-trawl accumulation factors.
