# Supplementary material for: Heavy-Metal Rankings Across Sediment Risk, Seafood Bioaccumulation and Dietary Hazard in the Maowei Sea, China
Source: Toxics. 2026 Sep 30;14(10):875. doi: 10.3390/toxics14100875 (PMC13709860; doi:10.3390/toxics14100875)
Supplement: Supplementary file 1 [file toxics-14-00875-s001.zip › toxics-4574334-supplementary.pdf]

Supplementary Materials

Heavy-metal rankings across sediment risk, seafood bioaccumulation and dietary hazard in the Maowei Sea, China

Enze Zhang, Lijiao Deng, Jun Zeng, Junxiang Lai, Hao Li*

* Corresponding author: leslieleehao@163.com

Text S1. Index formulae, denominators and class boundaries

Water quality index. WQI = Σ (Ci / Si), where Ci is the measured concentration of metal i and Si its Class I limit under GB 3097-1997 [1] (Cu 5, Pb 1, Zn 20, Cd 1, Cr 50, Hg 0.05, As 20 μg L^{-1}).

Geo-accumulation index [2]. $I_{geo} = \log_2[C / (1.5\ B)]$, where C is the measured sediment concentration and B the regional geochemical background [3] (Cu 15.8, Pb 28.9, Zn 75.8, Cd 0.09, Cr 39.9, Hg 0.029, As 7.8 mg kg^{-1}). Classes: $I_{geo} \leq 0$ unpolluted; 0–1 unpolluted to moderately polluted; 1–2 moderately polluted; 2–3 moderately to heavily polluted; 3–4 heavily polluted; 4–5 heavily to extremely polluted; > 5 extremely polluted.

Potential ecological risk [4]. $E_r = T_r \times C / B$ with toxic-response factors T_r of Zn 1, Cr 2, Cu 5, Pb 5, As 10, Cd 30 and Hg 40; RI = Σ E_r over the seven metals. Classes: $E_r < 40$ low, 40–80 moderate, 80–160 considerable, 160–320 high, ≥ 320 very high; RI < 150 low, 150–300 moderate, 300–600 considerable, ≥ 600 very high.

Mean effects-range-median quotient [5,6]. mERM-Q = (1/n) Σ (Ci / ERMi), with ERM values of Cu 270, Pb 218, Zn 410, Cd 9.6, Cr 370, Hg 0.71 and As 70 mg kg^{-1}. Classes: < 0.1, 9% incidence of toxicity; 0.1–0.5, 21%; 0.5–1.5, 49%; > 1.5, 76%.

Field bioaccumulation factor relative to water. BAF = C_{biota} (mg kg^{-1} fresh weight) / C_{water} (mg L^{-1}), where C_{biota} is the arithmetic mean of 12 station-level tissue results per taxon. In the main text C_{water} is the mean of all 27 Maowei Sea water stations, the area in which the animals were taken (trawl stations YM1 to YM12); the mean of the stations lying within 3 km of a trawl transect (6, 8, 9, 10, 11, 14, 15, 24, 25, 26) is used in the sensitivity comparison of Fig. S1. Because the animals were exposed through water, food and sediment simultaneously, this is a field tissue-to-water ratio in the sense of the USEPA bioaccumulation factor [7], not a laboratory bioconcentration factor.

Biota-sediment accumulation factor. BSAF = $C_{biota,dw}$ / $C_{sediment,dw}$, with $C_{biota,dw}$ = $C_{biota,fw}$ / (1 − moisture fraction), moisture fractions of 0.78 (crustaceans), 0.82 (bivalves) and 0.77 (fish), and $C_{sediment,dw}$ the mean of all 15 Maowei Sea sediment stations in the main text, or of the sediment stations within 3 km of a trawl transect (6, 8, 9, 10, 11, 15, 24, 26) in Fig. S1.

Estimated daily intake and target hazard quotient [7,8]. EDI = C × IR / BW; THQ = (EF × ED × IR × C) / (RfD × BW × AT), with EF = 365 d a^{-1}, ED = 70 a, AT = ED × 365 d, IR = 48.0 g d^{-1} (0.048 kg d^{-1} in the formula) [9], BW = 60 kg, and oral reference doses (mg kg^{-1} d^{-1}) of Zn 0.3, Cd 0.001, Cr 1.5 (as Cr(III)), Hg 1 × 10^{-4} (as methylmercury) and inorganic As 6 × 10^{-5} from IRIS [10]; Cu has no IRIS reference dose and the screening value 0.04 of the USEPA Regional Screening Level tables, derived from the drinking-water action level, is used [11,12]; Pb has no IRIS reference dose and the screening value

0.0035 derived from the 1993 JECFA provisional tolerable weekly intake (withdrawn in 2010) is used only to keep Pb in the sum. The inorganic fraction of total As, which was not measured, is carried as two scenarios, 10% (generic screening default) and 5% (motivated by measured values in Chinese seafood from other regions), with 2% and 1% as further sensitivity cases in Table S3. HI = Σ THQ. Results with the superseded As reference dose of 3×10^{-4} are given in Table S3.

Monte Carlo inputs. Ingestion rate lognormal (geometric mean 40 g d^{-1}, geometric standard deviation 1.8); body weight normal (60 ± 10 kg) sampled from the distribution truncated at 40 and 100 kg; tissue concentration normal, centred on the 12-station arithmetic mean for each taxon, with a coefficient of variation of 10% (analytical variation only); non-detects uniform between 0 and the detection limit; inorganic As fraction uniform between 1% and 10% (main prior, Table 5) or between 0.2% and 5.7% (alternative prior, Table S4); methylmercury fraction uniform between 70 and 95% (fish), 50 and 90% (crustaceans) and 30 and 70% (bivalves); 20,000 iterations with the same intake and body-weight draws for every taxon. The reported share of the leading metal is the median of its share of HI over the iterations in which it leads.

Table S1. Station-level data

(a) Water column, 27 stations (April 2022). NA, not recorded.

Station	Zone	Longitude (°E)	Latitude (°N)	Salinity	Cu (μg L^{-1})	Pb (μg L^{-1})	Zn (μg L^{-1})	Cd (μg L^{-1})	Cr (μg L^{-1})	Hg (μg L^{-1})	As (μg L^{-1})
1	Inner basin	108.4628	21.8933	18.0	0.93	0.062	9.6	0.065	0.2	0.1	1.4
2	Inner basin	108.4661	21.8814	17.8	1	0.015	6.8	0.039	0.2	0.096	1.4
3	Inner basin	108.4742	21.8719	13.8	0.85	0.015	6.4	0.28	0.2	0.094	1.2

Station	Zone	Longitude (°E)	Latitude (°N)	Salinity	Cu (μg L⁻¹)	Pb (μg L⁻¹)	Zn (μg L⁻¹)	Cd (μg L⁻¹)	Cr (μg L⁻¹)	Hg (μg L⁻¹)	As (μg L⁻¹)
4	Inner basin	108.4872	21.8672	13.8	1.2	0.015	16.9	0.1	0.2	0.093	2.4
5	Inner basin	108.4981	21.8642	14.1	0.58	0.015	5.6	0.089	0.22	0.089	1.2
6	Inner basin	108.5169	21.8633	14.3	0.36	0.015	13.8	0.047	0.2	0.087	1.4
7	Inner basin	108.5267	21.8761	14.2	1.4	0.015	14.6	0.065	0.17	0.039	1.1
8	Inner basin	108.5244	21.8592	12.5	0.58	0.015	5.4	0.092	0.45	0.04	1.3
9	Bay neck	108.5211	21.8439	16.6	0.23	0.065	9.7	0.045	0.2	0.035	1.4
10	Bay neck	108.5208	21.8317	18.1	0.65	0.015	6.9	0.27	0.94	0.04	1.6
11	Bay neck	108.5206	21.8214	18.4	0.64	0.015	11.2	0.076	0.72	0.035	1.7
12	Bay neck	108.5064	21.8247	14.7	0.47	0.015	13.4	0.017	0.2	0.041	1.7
13	Bay neck	108.5067	21.8339	14.2	0.46	0.22	9.8	0.005	0.59	0.047	1.5
14	Bay neck	108.5078	21.8444	17.2	1.3	0.18	14	0.042	0.2	0.043	1.5
15	Inner basin	108.5078	21.8556	13.3	0.1	0.015	13.7	0.044	0.2	0.047	1.5
16	Bay neck	108.4869	21.8358	11.3	0.1	0.015	10.1	0.22	0.53	0.056	1.6
17	Bay neck	108.4761	21.8406	10.1	1.3	0.015	16.7	0.24	0.44	0.074	1.2
18	Inner basin	108.4672	21.8506	8.8	0.86	0.15	13	0.11	0.2	0.076	1.8
19	Inner basin	108.4594	21.8608	7.5	1.2	0.015	10.3	0.064	0.2	0.073	1.2
20	Inner basin	108.4572	21.8703	6.2	0.83	0.015	11.2	0.063	0.43	0.084	1.5
21	Inner basin	108.6444	21.8783	< 2	1.7	0.015	16.3	0.077	1.7	0.062	1.7
22	Inner basin	108.6219	21.8769	< 2	0.71	0.015	10.8	0.031	0.71	0.062	1.5
23	Bay neck	108.6192	21.8386	11.6	0.28	0.015	14.5	0.047	0.2	0.072	1.7
24	Bay neck	108.5983	21.8464	14.9	0.83	0.041	14.1	0.044	0.83	0.073	1.3
25	Bay neck	108.5564	21.8431	14.8	0.45	0.088	10.5	0.063	0.2	0.03	1.6
26	Bay neck	108.5461	21.8075	17.7	0.75	0.015	13.8	0.062	0.2	0.035	1.6
28	Bay neck	108.6578	21.7803	26.3	0.64	0.015	15	0.088	0.2	0.058	1.1

(b) Surface-sediment concentrations, 15 stations. Concentrations are on a dry-weight basis; OC, organic carbon; NA, not recorded.

**Station**	**Zone**	**Cu (mg kg⁻¹)**	**Pb (mg kg⁻¹)**	**Zn (mg kg⁻¹)**	**Cd (mg kg⁻¹)**	**Cr (mg kg⁻¹)**	**Hg (mg kg⁻¹)**	**As (mg kg⁻¹)**	**OC (%)**	**Sulfide (mg kg⁻¹)**
2	Inner basin	40.6	27.7	114	0.44	34.8	0.063	12.5	0.72	8.5
3	Inner basin	18.6	18.6	48.7	0.68	36.3	0.027	5.5	0.49	9
4	Inner basin	5.3	8.4	19.1	0.044	39.1	0.067	3.2	0.10	7.6
6	Inner basin	15.6	19.9	49.1	0.12	48.6	0.026	9.4	0.45	0.74
8	Inner basin	16.7	20.2	46.4	0.45	32.5	0.064	7.5	0.53	0.98
9	Bay neck	17.8	20.7	58.2	0.073	37.4	0.026	13.9	0.78	6.4
10	Bay neck	18.5	22.3	67.8	0.061	32.1	0.06	11.7	0.74	2.1
11	Bay neck	7.03	17.5	34.2	0.25	16.5	0.007	5	0.14	1
13	Bay neck	16.9	25.4	57.1	0.13	28.4	0.042	9.4	0.65	1.5
15	Inner basin	33	27.4	88.8	0.27	51.5	0.05	13.2	1.60	5.2
17	Bay neck	32.7	12.7	44.6	0.12	60.3	0.046	9.8	0.83	5.2
19	Inner basin	7.1	13.1	36.4	0.44	7.3	0.054	4.4	0.33	NA
22	Inner basin	17	23.7	50.5	0.18	40	0.023	7.9	0.17	4.8
24	Bay neck	22.5	39.9	103	0.17	92.3	0.095	16.9	1.50	18
26	Bay neck	7.8	18.8	44.3	0.08	39.3	0.003	9.4	0.02	1.2

(c) Indices for the same 15 sediment stations: geo-accumulation index, ecological risk index and mean ERM quotient. NA, not recorded.

**Station**	**Zone**	**I_{geo} Cu**	**I_{geo} Pb**	**I_{geo} Zn**	**I_{geo} Cd**	**I_{geo} Cr**	**I_{geo} Hg**	**I_{geo} As**	**RI**	**mERM-Q**
2	Inner basin	0.78	-0.65	0.00	1.70	-0.78	0.53	0.10	270	0.138
3	Inner basin	-0.35	-1.22	-1.22	2.33	-0.72	-0.69	-1.09	283	0.080
4	Inner basin	-2.16	-2.37	-2.57	-1.62	-0.61	0.62	-1.87	117	0.051
6	Inner basin	-0.60	-1.12	-1.21	-0.17	-0.30	-0.74	-0.32	99	0.083
8	Inner basin	-0.51	-1.10	-1.29	1.74	-0.88	0.56	-0.64	259	0.086
9	Bay neck	-0.41	-1.07	-0.97	-0.89	-0.68	-0.74	0.25	90	0.092

Station	Zone	I_{geo} Cu	I_{geo} Pb	I_{geo} Zn	I_{geo} Cd	I_{geo} Cr	I_{geo} Hg	I_{geo} As	RI	mERM-Q
10	Bay neck	-0.36	-0.96	-0.75	-1.15	-0.90	0.46	0.00	130	0.097
11	Bay neck	-1.75	-1.31	-1.73	0.89	-1.86	-2.64	-1.23	106	0.049
13	Bay neck	-0.49	-0.77	-0.99	-0.05	-1.08	-0.05	-0.32	125	0.086
15	Inner basin	0.48	-0.66	-0.36	1.00	-0.22	0.20	0.17	195	0.127
17	Bay neck	0.46	-1.77	-1.35	-0.17	0.01	0.08	-0.26	132	0.095
19	Inner basin	-1.74	-1.73	-1.64	1.70	-3.04	0.31	-1.41	232	0.054
22	Inner basin	-0.48	-0.87	-1.17	0.42	-0.58	-0.92	-0.57	114	0.081
24	Bay neck	-0.07	-0.12	-0.14	0.33	0.62	1.13	0.53	229	0.166
26	Bay neck	-1.60	-1.21	-1.36	-0.75	-0.61	-3.86	-0.32	51	0.068

Table S2. Sensitivity of the biota-sediment accumulation factor to the assumed moisture content

BSAF against the Maowei Sea sediment mean, recomputed with the moisture fraction of each group lowered and raised by five percentage points. Metals below detection are omitted.

Species	Group	Metal	Moisture −5 pp	Base moisture	Moisture +5 pp	Base (%)
Metapenaeus joyneri	Crustacean	Cu	1.06	1.30	1.69	78
Metapenaeus joyneri	Crustacean	Zn	0.68	0.84	1.08	78
Metapenaeus joyneri	Crustacean	Cd	0.27	0.33	0.43	78
Metapenaeus joyneri	Crustacean	Hg	0.68	0.84	1.08	78
Eriocheir sp.	Crustacean	Cu	3.59	4.40	5.70	78
Eriocheir sp.	Crustacean	Zn	1.75	2.14	2.77	78
Eriocheir sp.	Crustacean	Cd	0.62	0.76	0.98	78
Eriocheir sp.	Crustacean	Hg	1.62	1.98	2.57	78
Eriocheir sp.	Crustacean	As	0.13	0.16	0.20	78
Geloina erosa	Bivalve	Cu	0.33	0.42	0.58	82
Geloina erosa	Bivalve	Pb	0.02	0.03	0.04	82
Geloina erosa	Bivalve	Zn	1.49	1.90	2.64	82
Geloina erosa	Bivalve	Cd	3.53	4.51	6.25	82

Species	Group	Metal	Moisture −5 pp	Base moisture	Moisture +5 pp	Base (%)
Geloina erosa	Bivalve	Cr	0.01	0.02	0.02	82
Geloina erosa	Bivalve	Hg	1.20	1.53	2.12	82
Geloina erosa	Bivalve	As	0.11	0.14	0.20	82
Crassostrea sp.	Bivalve	Cu	2.47	3.16	4.37	82
Crassostrea sp.	Bivalve	Pb	0.08	0.10	0.14	82
Crassostrea sp.	Bivalve	Zn	5.31	6.78	9.39	82
Crassostrea sp.	Bivalve	Cd	3.35	4.28	5.92	82
Crassostrea sp.	Bivalve	Cr	0.00	0.01	0.01	82
Crassostrea sp.	Bivalve	Hg	0.70	0.89	1.24	82
Crassostrea sp.	Bivalve	As	0.11	0.14	0.19	82
Meretrix meretrix	Bivalve	Cu	0.33	0.42	0.58	82
Meretrix meretrix	Bivalve	Pb	0.04	0.06	0.08	82
Meretrix meretrix	Bivalve	Zn	0.89	1.14	1.58	82
Meretrix meretrix	Bivalve	Cd	3.72	4.75	6.58	82
Meretrix meretrix	Bivalve	Cr	0.01	0.02	0.03	82
Meretrix meretrix	Bivalve	Hg	0.60	0.77	1.06	82
Meretrix meretrix	Bivalve	As	0.39	0.50	0.69	82
Lateolabrax maculatus	Fish	Cu	0.10	0.12	0.15	77
Lateolabrax maculatus	Fish	Zn	0.28	0.34	0.43	77
Lateolabrax maculatus	Fish	Cd	0.15	0.19	0.24	77
Lateolabrax maculatus	Fish	Hg	1.31	1.60	2.04	77
Chelon affinis	Fish	Cu	0.09	0.11	0.14	77
Chelon affinis	Fish	Zn	0.32	0.39	0.49	77
Chelon affinis	Fish	Cd	0.15	0.19	0.24	77
Chelon affinis	Fish	Hg	0.90	1.10	1.40	77
Chelon affinis	Fish	As	0.09	0.11	0.14	77

Table S3. Sensitivity of the hazard index to ingestion rate, body weight, the inorganic-As fraction and the As reference dose

(a) Ingestion rate, body weight and As reference dose, at an inorganic-As fraction of 10%. All columns use the 2025 IRIS reference dose for inorganic As (6×10^{-5} mg kg^{-1}

d⁻¹) except the last, which repeats the base case (48 g d⁻¹, 60 kg) with the superseded 1991 value (3×10^{-4}).

Species	Group	HI, 30 g d⁻¹, 60 kg	HI, 48 g d⁻¹, 60 kg	HI, 48 g d⁻¹, 70 kg	HI, 100 g d⁻¹, 60 kg	IR at HI = 1, fAs 10% (g d⁻¹)	HI, 48 g d⁻¹, 60 kg, As RfD 1991
Metapenaeus joyneri	Crustacean	0.22	0.35	0.30	0.73	137	0.24
Eriocheir sp.	Crustacean	0.65	1.04	0.90	2.18	46	0.70
Geloina erosa	Bivalve	0.42	0.67	0.58	1.40	71	0.42
Crassostrea sp.	Bivalve	0.62	0.99	0.85	2.06	48	0.75
Meretrix meretrix	Bivalve	0.90	1.44	1.23	2.99	33	0.54
Lateolabrax maculatus	Fish	0.18	0.30	0.25	0.62	162	0.19
Chelon affinis	Fish	0.27	0.43	0.37	0.90	112	0.18

(b) Inorganic-As fraction. Because the As quotient is linear in the fraction, the fraction above which As overtakes the next-largest quotient and the fraction at which HI reaches 1 at the base intake (48 g d⁻¹, 60 kg) are solved exactly; a fraction above 20% is reported as "> 20%".

Species	Group	HI, 48 g d⁻¹, 60 kg, fAs 5%	HI, 100 g d⁻¹, 60 kg, fAs 5%	HI, 48 g d⁻¹, 60 kg, fAs 2%	HI, 48 g d⁻¹, 60 kg, fAs 1%	Leading metal at fAs 10 / 5 / 2 / 1%	fAs above which As leads (metal it overtakes)	fAs at which HI = 1 (48 g d⁻¹, 60 kg)	IR at HI = 1, fAs 5% (g d⁻¹)
Metapenaeus joyneri	Crustacean	0.28	0.59	0.24	0.23	As / Cu / Cu / Cu	8.0% (Cu)	> 20%	170
Eriocheir sp.	Crustacean	0.83	1.73	0.70	0.66	As / Cu / Cu / Cu	8.4% (Cu)	9.0%	58
Geloina erosa	Bivalve	0.51	1.07	0.42	0.39	As / As / Cd / Cd	4.8% (Cd)	> 20%	93
Crassostrea sp.	Bivalve	0.84	1.75	0.75	0.71	As / Cu / Cu / Cu	6.8% (Cu)	10.3%	57
Meretrix meretrix	Bivalve	0.88	1.82	0.54	0.43	As / As / As / Cd	1.4% (Cd)	6.1%	55
Lateolabrax maculatus	Fish	0.23	0.48	0.19	0.18	As / Hg / Hg / Hg	9.6% (Hg)	> 20%	209
Chelon affinis	Fish	0.28	0.58	0.18	0.15	As / As / Hg / Hg	2.9% (Hg)	> 20%	174

Table S4. Monte Carlo summary under the alternative inorganic-As prior

Same simulation as Table 5 of the main text (20,000 iterations, same random seed and sampling order) with the inorganic-As fraction drawn from a uniform distribution between 0.2 and 5.7%, the range measured in eleven cultured shellfish species of Sanmen Bay [13], instead of 1 to 10%. Neither prior is a measurement for the taxa sampled here.

**Species**	**Group**	**HI median**	**HI 95th percentile**	**Frequency HI > 1**	**Frequency any THQ > 1**	**Most frequent leading metal (frequency; median share when leading)**	**Frequency As leads**
Metapenaeus joyneri	Crustacean	0.19	0.54	0.5%	0.0%	Cu (94%; 47%)	6%
Eriocheir sp.	Crustacean	0.58	1.58	18.8%	2.5%	Cu (100%; 51%)	0%
Geloina erosa	Bivalve	0.33	0.91	3.6%	0.1%	Cd (83%; 39%)	17%
Crassostrea sp.	Bivalve	0.62	1.69	21.9%	0.3%	Cu (79%; 29%)	0%
Meretrix meretrix	Bivalve	0.49	1.50	15.1%	3.8%	As (78%; 57%)	78%
Lateolabrax maculatus	Fish	0.15	0.42	0.2%	0.0%	Hg (94%; 63%)	6%
Chelon affinis	Fish	0.16	0.47	0.3%	0.0%	As (60%; 53%)	60%

Fig. S1. Whole-bay versus near-trawl accumulation factors

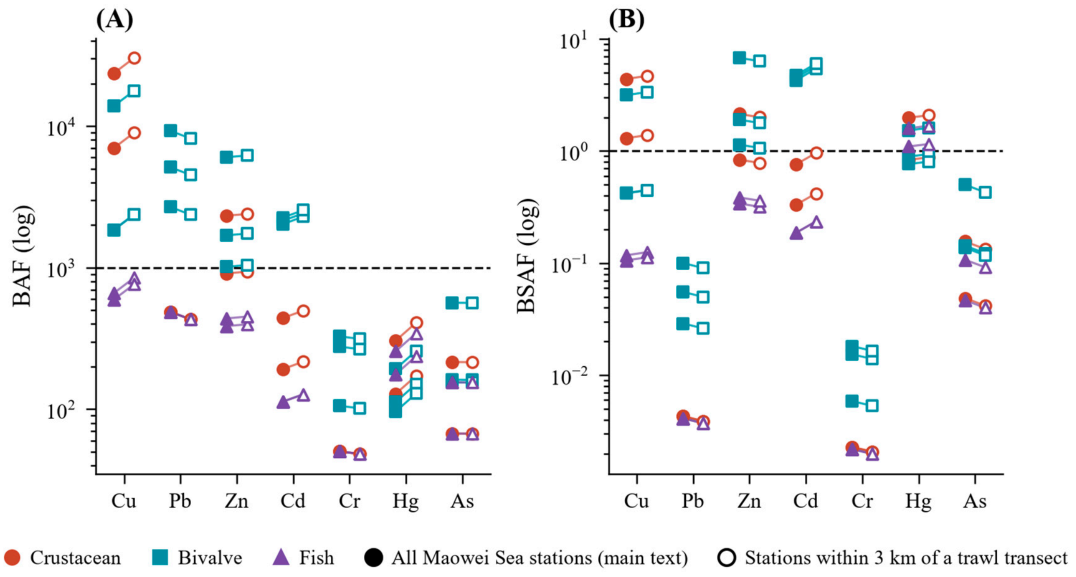

Fig. S1. Field bioaccumulation factors relative to water (A) and biota-sediment accumulation factors (B) computed with the whole-bay denominators used in the main text (filled symbols; 27 water and 15 sediment stations) and with the stations lying within 3 km of a trawl transect (open symbols; water stations 6, 8, 9, 10, 11, 14, 15, 24, 25, 26; sediment stations 6, 8, 9, 10, 11, 15, 24, 26). Ratios of the near-trawl mean to the whole-bay mean were: Cu 0.78 (water), 0.94 (sediment); Pb 1.13 (water), 1.11 (sediment); Zn 0.97 (water), 1.07 (sediment); Cd 0.89 (water), 0.79 (sediment); Cr 1.04 (water), 1.10 (sediment); Hg 0.75 (water), 0.95 (sediment); As 1.00 (water), 1.17 (sediment). Individual BSAF values change by between 0.86- and 1.27-fold and individual BAF values by between 0.88- and 1.34-fold. No metal crosses the BAF = 1000 or BSAF = 1 reference line under either denominator, and the metal with the highest BSAF is the same in all seven taxa. The highest BAF changes in one taxon: in *Geloina erosa* Pb leads against the whole-bay water mean and Cd against the near-trawl mean.

References

1. GB 3097-1997. *Sea Water Quality Standard*; State Environmental Protection Administration: Beijing, China, 1997.

2. Müller, G. Schwermetalle in den Sedimenten des Rheins: Veränderungen seit 1971. *Umschau* **1979**, *79*, 778–783.

3. Bi, W.-N.; Hong, M.; Peng, M.-W.; Lan, W.-L. Assessment on the pollution and risk of heavy metals in surface sediments of Guangxi offshore from 2005 to 2020. *Mar. Environ. Sci.* **2022**, *41*, 416–422. (In Chinese with English abstract) https://doi.org/10.12111/j.mes2021-x-0046

4. Håkanson, L. An ecological risk index for aquatic pollution control. A sedimentological approach. *Water Res.* **1980**, *14*, 975–1001. https://doi.org/10.1016/0043-1354(80)90143-8

5. Long, E.R.; MacDonald, D.D.; Smith, S.L.; Calder, F.D. Incidence of adverse biological effects within ranges of chemical concentrations in marine and estuarine sediments. *Environ. Manag.* **1995**, *19*, 81–97. https://doi.org/10.1007/BF02472006

6. Long, E.R.; MacDonald, D.D. Recommended uses of empirically derived, sediment quality guidelines for marine and estuarine ecosystems. *Hum. Ecol. Risk Assess.* **1998**, *4*, 1019–1039. https://doi.org/10.1080/10807039891284956

7. U.S. Environmental Protection Agency. *Guidance for Assessing Chemical Contaminant Data for Use in Fish Advisories, Volume 2: Risk Assessment and Fish*

Consumption Limits, 3rd ed.; EPA 823-B-00-008; Office of Water: Washington, DC, USA, 2000.

8. U.S. Environmental Protection Agency. *Risk Assessment Guidance for Superfund, Volume I: Human Health Evaluation Manual (Part A)*; EPA/540/1-89/002; Office of Emergency and Remedial Response: Washington, DC, USA, 1989.

9. Wang, Q.; Liu, S.; Wang, H.; Su, C.; Liu, A.; Jiang, L. Consumption of aquatic products and meats in Chinese residents: A nationwide survey. *Front. Nutr.* **2022**, *9*, 927417. https://doi.org/10.3389/fnut.2022.927417

10. U.S. Environmental Protection Agency. *IRIS Toxicological Review of Inorganic Arsenic (Final Report)*; Integrated Risk Information System: Washington, DC, USA, 2025. Available online: https://iris.epa.gov/ChemicalLanding/&substance_nmbr=278 (accessed on 3 September 2026).

11. U.S. Environmental Protection Agency. Regional Screening Levels (RSLs): Generic Tables. Available online: https://www.epa.gov/risk/regional-screening-levels-rsls-generic-tables (accessed on 21 September 2026).

12. Taylor, A.A.; Tsuji, J.S.; McArdle, M.E.; Adams, W.J.; Goodfellow, W.L., Jr. Recommended reference values for risk assessment of oral exposure to copper. *Risk Anal.* **2023**, *43*, 211–218. https://doi.org/10.1111/risa.13906

13. Ran, M.; Shi, Y.; Wu, D.; Ye, H.; Feng, D.; Huang, D.; Li, S.; Fang, C. Characteristics of arsenic speciation in mainly cultured shellfish from Sanmen Bay, Zhejiang Province, China. *Mar. Pollut. Bull.* **2023**, *197*, 115793. https://doi.org/10.1016/j.marpolbul.2023.115793
